# Multitable Methods for Microbiome Data Integration

**DOI:** 10.3389/fgene.2019.00627

**Published:** 2019-08-28

**Authors:** Kris Sankaran, Susan P. Holmes

**Affiliations:** ^1^Mila, Universite de Montréal, Montréal, QC, Canada; ^2^Department of Statistics, Stanford University, Stanford, CA, United States

**Keywords:** microbiome, data integration, multiomics, dimensionality reduction, heterogeneity

## Abstract

The simultaneous study of multiple measurement types is a frequently encountered problem in practical data analysis. It is especially common in microbiome research, where several sources of data—for example, 16s-rRNA, metagenomic, metabolomic, or transcriptomic data–can be collected on the same physical samples. There has been a proliferation of proposals for analyzing such multitable microbiome data, as is often the case when new data sources become more readily available, facilitating inquiry into new types of scientific questions. However, stepping back from the rush for new methods for multitable analysis in the microbiome literature, it is worthwhile to recognize the broader landscape of multitable methods, as they have been relevant in problem domains ranging across economics, robotics, genomics, chemometrics, and neuroscience. In different contexts, these techniques are called data integration, multi-omic, and multitask methods, for example. Of course, there is no unique optimal algorithm to use across domains—different instances of the multitable problem possess specific structure or variation that are worth incorporating in methodology. Our purpose here is not to develop new algorithms, but rather to 1) distill relevant themes across different analysis approaches and 2) provide concrete workflows for approaching analysis, as a function of ultimate analysis goals and data characteristics (heterogeneity, dimensionality, sparsity). Towards the second goal, we have made code for all analysis and figures available online at https://github.com/krisrs1128/multitable_review.

Most methods in statistics expect data to be available as a single table. To a researcher confronted with multiple sources of data, it might therefore seem most natural to either analyze each source separately, one at a time, or else combine all data into a single, unified table. However, neither of these approaches is entirely satisfactory. First, many scientific problems can only be answered by collecting several complementary measurement types. Indeed, the situation is analogous to using many types of sensors to study a single system from many perspectives. Further, while in certain supervised problems, it is enough to predict a single measurement of interest, with other sources collected primarily to provide better features, there are often additional relational components to the analysis: how do different types of measurements co-vary with one another? Here, it is of interest to provide a representation of the data that facilitates comparisons across tables, rather than just comparing each table with a single response of interest. This richer scientific question motivates the development of methods distinct from those used to analyze a single measurement type at a time.

For more concrete motivation, we consider data from the WELL-China study, which is focused on the relationships between various indicators of wellness (Min et al., 2019). In this study, 1,969 individuals^1^ underwent clinical examinations, filled out wellness surveys (covering topics such as exercise, sleep, diet, and mental health, for example), and provided stool samples, used for 16s-rRNA sequencing and metabolomic analysis. To date, 16s-rRNA sequencing data are available for 221 of these participants. Evidently, various interesting relational questions can be investigated using this data source.

For the purpose of illustration, we focus on one relatively narrow question that can be addressed using these data: How is the distribution of lean and fat mass across the body related to patterns of microbial abundance? The measurement types most relevant in this analysis are DEXA scans and 16s-rRNA sequencing abundances. DEXA scans use relative X-ray absorption to gauge the amount of lean and fat body mass within a region of the body being scanned. We have access to these lean and fat body mass measurements at several body sites—arms, legs, trunk, etc.—along with related body type variables, like height, age, and android and gynoid fat measurements. In total, there are 36 of these variables. 16s-rRNA sequencing is a technology for gauging the abundance of different bacterial species in the gut by counting the alignments of reads to the 16s-rRNA gene, a component of all bacterial genomes with enough variation to allow discrimination between different individual species. We have counts associated with 2,565 species across 181 genera, though the vast majority are present in low abundances.

This question of the relationship between lean and fat mass distribution (informally, “body type”) and the microbiome is motivated by findings that certain taxonomic groups are over- or underrepresented as a function of an individual’s body mass index (BMI) (Ley et al., 2005; Ley et al., 2006; Turnbaugh et al., 2009; Ley, 2010). Further, since the distribution of fat is often more related to underlying biological mechanisms than overall body mass (Matsuzawa, 2008), and since this distribution is mediated by specific metabolic pathways, there is reason to suspect that a joint analysis of DEXA and 16s-rRNA microbial abundance data might yield a more complete view of the relationship between the microbiome and body type.

We use this motivating dataset in the examples that follow. Additional numerical examples, for methods only discussed abstractly in this review, are available in the github repository associated with this paper.

## Classical Multivariate Methods

Methods from classical multivariate statistics are a mainstay of single-table microbiome data analysis, so it is natural to revisit them before surveying extensions to the multitable setting. Here, we explore a few of the classically studied multitable methods that fit nicely into the modern microbiome data analysis toolbox. We first describe a naive approach based on Principal Components Analysis (PCA)—naive because it lifts a single-table method to the multiple table setting without any special considerations—before studying approaches that directly characterize covariation across several tables: Canonical Correlation Analysis (CCA), Multiple Factor Analysis (MFA), and Principal Component Analysis with Instrumental Variables (PCA-IV).

The earliest multitable method (CCA) was published in 1936, motivated by the problem of relating prices of groups of commodities (Hotelling, 1936). There are two notable aspects of data analysis in this classical paradigm that no longer hold in modern statistics,

- Even when many samples could be collected, there were typically only a few features for each sample, and it was straightforward to study all of them simultaneously. It is now possible to automatically collect a large number of features for each observation (or subject).
- Before electronic computers had been invented, it was important that all statistical quantities be easy to calculate, typically necessitating analytical formulas for parameter estimates. This is no longer an important limitation due to modern computation.

These changes have driven the development of high-dimensional methods and facilitated the adoption of iterative, more computationally intensive approaches.

Nonetheless, it is worth reviewing these original approaches, both to understand the context for many modern techniques and to have an easy starting point for practical data analysis. Indeed, these more established methods tend to be the most readily available through statistical computing packages and can provide a benchmark with which to compare more elaborate, modern methods.

### PCA

The simplest approach to dealing with multiple tables is to combine them into one and apply a single-table method, for example, PCA. That is, write

$$X=\left[X^{\left(1\right)}\left|\;\ldots \;\right|X^{\left(L\right)}\right]\;\in {\mathbb{R}}^{n\times p},$$

where $p=\sum_{l=1}^{L}\mathrm{pl}$, and compute the SVD *X* = *UDV^^* ^T^. The *K*-principal component directions are the first *K* columns *v*_1 _,…, *v_K_*, while the associated scores are reweighted rows *d*_1_ *u*_1_,…, *d*_K_ *u_K_*. We call this method concatenated PCA.

While this does not account for the multitable structure of the data, it does accomplish two goals:

- Through the principal component scores, it provides a visualization of the relationships between samples, based on all features.
- Through the principal component directions, it gives a way of relating features within and across the multiple tables.

However, two drawbacks of this approach are worth noting:

- It does not provide a summary of the relationship between the sets of variables defining the tables—it can only relate pairs of variables.
- If some tables have many more variables than others, they can dominate the resulting ordination.

These limitations are addressed by CCA and MFA, discussed in sections CCA and MFA, respectively.

We provide one geometric and one statistical motivation for PCA. The geometric motivation is that, if each row *x_i_* of *X* is viewed as a point in *p*-dimensional space, then the principal component directions provide the best *K*-dimensional approximation to the data. The second interpretation is that PCA finds a low-dimensional representation of the *x_i_* such that the resulting points have maximal variance. Qualitatively, this is a desirable property, because it means that the simpler representation preserves most of the variation present in the original data.

PCA is a very widely used technique, and some standard references include Mardia et al. (1980), Friedman et al. (2001), and Pagés (2014). Nonetheless, it is not ideal in the multitable setting.

#### Example

**Figure 1:**
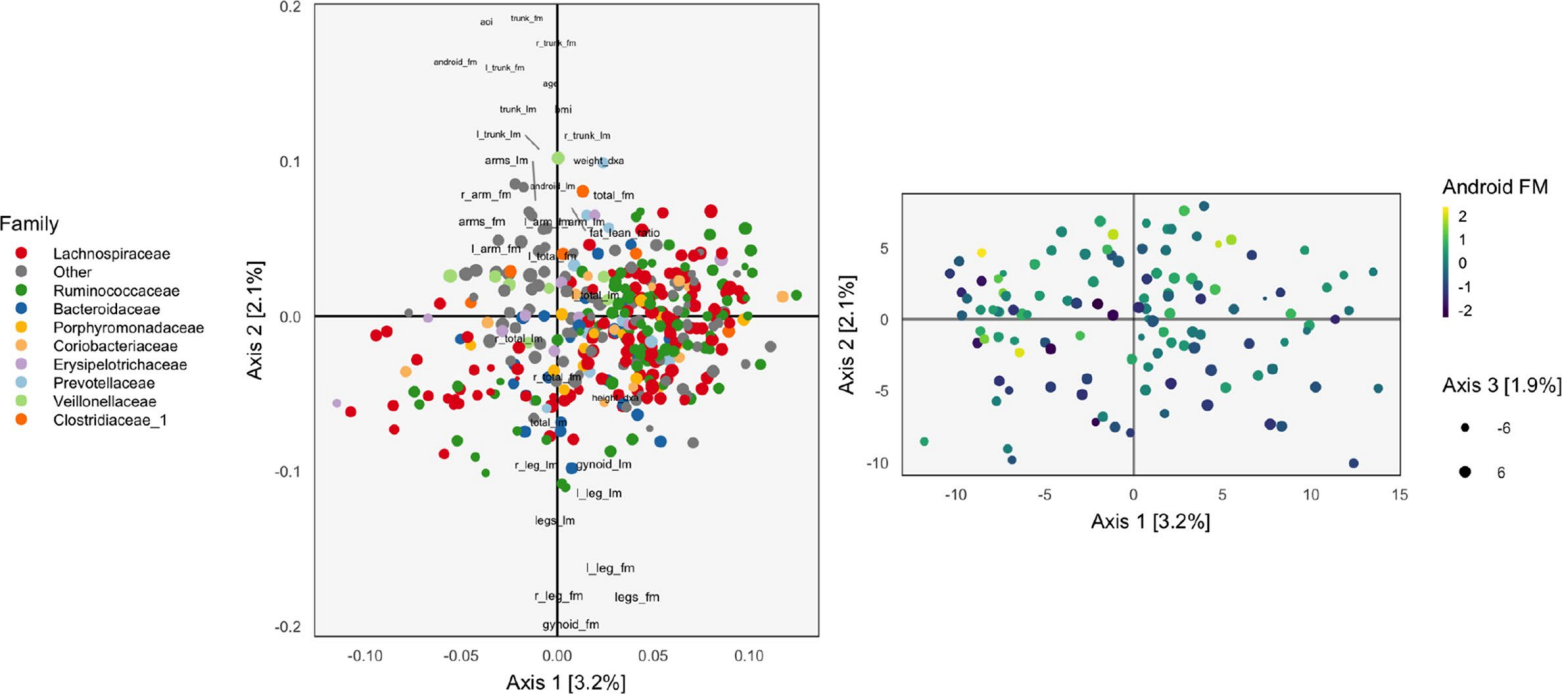
The loadings (left) and scores (right) obtained by applying Principal Components Analysis (PCA) to the combined body composition and microbial abundance data. For the loadings, species are points, and are shaded in by taxonomic family. Body composition variables are plotted as text. The size of points and words measures the contribution of the third PC dimension. For scores, each point corresponds to a sample.

**Figure 1** illustrates this approach on body composition and bacterial abundance data from the WELL-China study. Note that we have subsetted to only women, since men and women have very different body compositions, and we have slightly more data for women. Further, the 16s-rRNA data have been variance stabilized according to the methodology proposed in Anders and Huber (2010) and filtered to only those species that have count ≥5 in at least 7% of samples.

The left panel of **Figure 1** displays the loadings associated with this concatenated PCA approach, where body composition (36 columns) and 16s-rRNA abundances (372 columns) were combined into one dataset (408 columns). Columns associated with bacterial species are displayed as points, shaded by taxonomic family, while columns associated with body composition variables are labeled with text. Note that the fraction of variance explained by each axis is on the order of a few percent—this is to be expected, considering that the baseline proportion would be $\frac{1}{408}\approx 0.25\%$ in the orthogonal case.

Most body composition variables lie close to the vertical axis, in a direction approximately orthogonal to the main direction of variation among species. Columns that are highly correlated—e.g., right (R) and left (L) leg fat mass (FM)—have loadings nearly equal to one another. Among species, the most notable pattern is the concentration of Ruminococcaceae on the right.

To identify relationships between species and body composition variables, it would be of interest to isolate those species with large contributions along the axis defined by linking the center of the variables and the origin. Relatively few such species stand out, though note that there is nothing in this algorithm’s objective that would seek covariation across tables directly, so the fact that such associations seem weak with respect to the top two principal components does not mean such relationships do not exist.

We can study individual samples with respect to these loadings, by plotting their projections onto the top two principal components. This is the content of the right panel of **Figure 1**, which displays samples in the same positions, but shaded by android (i.e., abdominal) fat mass. This shading confirms the observations from the loadings directly using observed data. Indeed, the increasing android fat mass among samples in the top of the scores in that panel exactly corresponds to the fact that related variables lie at the top in the left panel.

In this approach, the loadings provide a description of the relationship between variables across datasets. Further, scores summarize variation in samples across multiple datasets. Hence, this heuristic is a natural first step in analyzing multiple table data. However, considering the difficulty in directly interpreting the covariation across datasets, as well as the method’s failure to use any sense of covariation in the dimensionality reductions strategy, suggests that this method should not be the last step of an analysis workflow. Nevertheless, we now have a baseline with which to compare the more elaborate methods of subsequent sections.

### CCA

CCA is a close relative of PCA, designed to compare sets of features across tables. Like PCA, it provides low-dimensional representations of observations, but it also allows comparisons at the table level. Suppose for now that there are only two tables of interest, $X\in {\mathbb{R}}^{n\times p_{1}}$ and $Y\in {\mathbb{R}}^{n\times p_{2}}$. Let ${\hat{\sum }}_{\mathrm{XX}},{\hat{\sum }}_{\mathrm{YY}}$, and ${\hat{\sum }}_{\mathrm{XY}}$ be the associated covariance estimates. Take the SVD, ${\hat{\sum }}_{{}_{\mathrm{XX}}}^{-\frac{1}{2}}{\hat{\sum }}_{\mathrm{XY}}{\hat{\sum }}_{{}_{\mathrm{YY}}}^{-\frac{1}{2}}=\tilde{U}D{\tilde{V}}^{T}$. The canonical correlation directions associated with the two tables are $u_{k}\sum_{{}_{\mathrm{XX}}}^{-\frac{1}{2}}{\tilde{u}}_{k}\in {\mathbb{R}}^{p_{1}}$ and $v_{k}=\sum_{{}_{\mathrm{YY}}}^{-\frac{1}{2}}{\tilde{v}}_{k}\in {\mathbb{R}}^{p_{2}}$. These directions give two sets of low-dimensional representations for each sample, one for each table: $z_{k}^{\left(1\right)}=Xu_{k}\in {\mathbb{R}}^{n}\text{and }z_{k}^{\left(2\right)}=Yv_{k}\in {\mathbb{R}}^{n}$. If the two tables are closely related, then the $z_{k}^{\left(1\right)}$ and $z_{k}^{\left(2\right)}$ will be very correlated. The singular values *d_k_* are called the canonical correlation coefficients. Like the eigenvalues in PCA, they characterize the amount of covariation across tables that can be captured by each additional pair of directions.

As with PCA, there are many ways to view this procedure—here we discuss geometric, statistical, and probabilistic interpretations. Unlike the geometric interpretation of PCA, the geometric interpretation for CCA identifies point locations with features, not samples. Specifically, the columns of *X* and *Y* are thought of as points in ℝ*^n^*. Consider two subspaces spanning the columns of *X* and *Y*, respectively. These subspaces correspond to the linear combinations of features within each table. Place two ellipses on the respective subspaces, centered at the origin and with size and shape depending on the within-table covariances ${\hat{\sum }}_{\mathrm{XX}}$ and ${\hat{\sum }}_{\mathrm{YY}}$. The first canonical correlation directions are the pair of points, one lying on each ellipse, such that the angle from the origin to those two points is smallest. In this sense, it finds a pair of variance-constrained linear combinations of features within the two tables such that the two combinations appear “close” to one another. The second pair of canonical correlation directions identify a pair of points with a similar interpretation, except they are required to be orthogonal to the first pair, with respect to the inner product induced by the covariances in each table.

For a statistical interpretation, the idea of CCA is to find the low-dimensional representations of the two tables with maximal covariance—this is analogous to the maximum variance interpretation. Formally, rows of the two tables are imagined to be i.i.d. draws from ℙ*^XY^*, which has marginals ℙ*^X^* and ℙ*^Y^*. Consider arbitrary linear combinations $z_{i}^{\left(1\right)}(u)=u^{T}x_{i}$ and $z_{i}^{\left(2\right)}(v)=v^{T}y_{i}$ of samples from the two tables. The first pair of CCA directions $u_{i}^{*}$ and $v_{i}^{*}$ are chosen to optimize

(1) $$\begin{array}{l} \underset{u\in {\mathbb{R}}^{p_{1}},v\in {\mathbb{R}}^{p_{2}}}{\mathrm{maximize}}\;{\text{Cov}}_{{\mathbb{P}}^{\mathrm{XY}}}\left[z_{i}^{\left(1\right)}(u),z_{i}^{\left(2\right)}(v)\right]\\ {\text{subject to Var}}_{{\mathbb{P}}^{X}}\left(z_{i}^{\left(1\right)}(u)\right)=1\\ \;{\text{Var}}_{{\mathbb{P}}^{Y}}\left(z_{i}^{\left(2\right)}(v)\right)=1 \end{array}$$

To produce subsequent directions, the same optimization is performed, but with the additional constraint that the directions must be orthogonal to all the previous directions identified for that table. Of course, in actual applications, we estimate these covariances and variances empirically.

This perspective makes it easy to derive the algorithm given at the start of this section. The empirical version of the optimization problem (1) is

(2) $$\begin{array}{l} \underset{u\in {\mathbb{R}}^{p_{1}},v\in {\mathbb{R}}^{p_{2}}}{\mathrm{maximize}}\;u^{T}{\hat{\sum }}_{\mathrm{XY}}v\\ \text{subject to }u^{T}{\hat{\sum }}_{\mathrm{XX}}u=1\\ \;v^{T}{\hat{\sum }}_{\mathrm{YY}}v=1. \end{array}$$

Consider the transformed data, $\tilde{u}={\hat{\sum }}_{\mathrm{XX}}^{\frac{1}{2}}u$ and $\tilde{v}={\hat{\sum }}_{\mathrm{YY}}^{\frac{1}{2}}v$. The optimization can be now be expressed as

(3) $$\begin{array}{l} \underset{\tilde{u}\in {\mathbb{R}}^{p_{1}},\tilde{\upsilon }\in {\mathbb{R}}^{p_{2}}}{\mathrm{maximize}}\;{\tilde{u}}^{T}{\hat{\sum }}_{\mathrm{XX}}^{-\frac{1}{2}}{\hat{\sum }}_{\mathrm{XY}}{\hat{\sum }}_{\mathrm{YY}}^{-\frac{1}{2}}\tilde{v}\\ \text{such that }||\tilde{u}|{|}_{2}^{2}=1\\ \;||\tilde{v}|{|}_{2}^{2}=1. \end{array}$$

The optimal ${\tilde{u}}_{1}$ nd ${\tilde{v}}_{1}$ for this problem are well known—they are exactly the first left and right eigenvectors of ${\hat{\sum }}_{\mathrm{XX}}^{\frac{1}{2}}{\hat{\sum }}_{\mathrm{XY}}{\hat{\sum }}_{\mathrm{YY}}^{-\frac{1}{2}}=\tilde{U}D{\tilde{V}}^{T}$, respectively.

A probabilistic interpretation of this procedure views it as estimating the factors in an implicit latent variable model. In particular, (Bach and Jordan, 2005) supposes that *x_i_* and *y_i_* are drawn i.i.d. from the model,

$$\begin{array}{l} \xi_{i}:=(\xi_{i}^{S},\xi_{i}^{x},\xi_{i}^{y})∼N(0,\mathrm{Id})\\ \;x_{i}|\xi_{i}∼N(\mu_{x}+W_{X}\xi_{i}^{S}+B_{X}\xi_{i}^{x},I_{d})\\ \;y_{i}|\xi_{i}∼N(\mu_{Y}+W_{Y}\xi_{i}^{S}+B_{Y}\xi_{i}^{y},I_{d}) \end{array}$$

That is, each sample is associated with a *d*-dimensional latent variable *ξ_i_*, drawn from a spherical normal prior. A few of the coordinates of these latent variables, $\xi_{i}^{s}$, contribute to shared structure, through *W_X_* and *W_Y_*. The remaining coordinates model table-specific structure, through *B_X_* and *B_Y_*. It can be shown that the posterior expectations of the latent $\xi_{i}^{s}$ given the observed tables must lie on the subspace defined by the CCA directions.

#### Example

**Figure 2:**
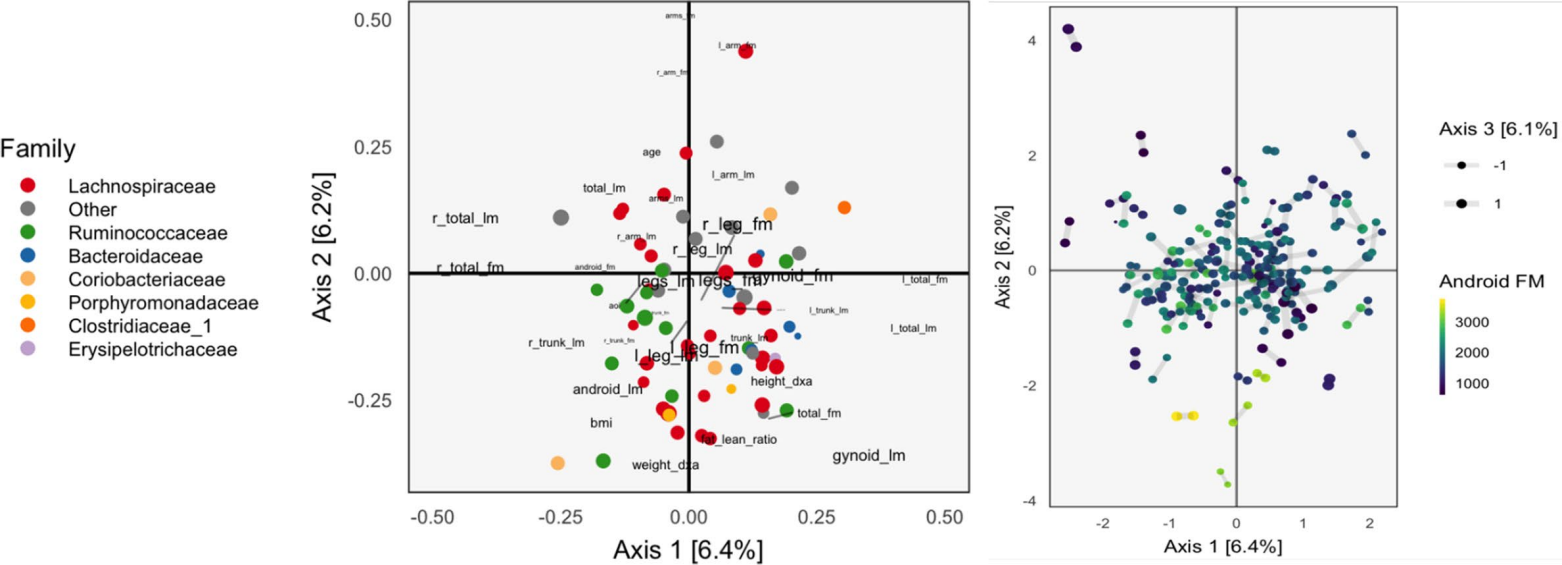
The Canonical Correlation Analysis (CCA) analog of the PCA biplot in **Figure 1**, obtained by applying CCA to the combined body composition and microbial abundance data. Since each sample is associated with a pair of scores, one from each table, we use a different symbol to represent the scores: two points joined by an edge, where each point gives the score from one of the tables. Aside from this exception, the PCA biplot interpretation still applies. The higher the CCA objective, the shorter the links between pairs. The first two CCA dimensions suggest smooth variation across samples, according to amount of android fat mass.

We next apply CCA to the WELL-China body composition and microbiome data, with particular interest in how the results compare with those of section Example. We provide analogous loadings and scores plots in **Figure 2**. However, note that the data are not quite the same between the two analysis—we have filtered down to species passing a filter, which reduces the number of species to 66, from 2,565. This very aggressive filtering is necessary because CCA requires estimation of covariances matrices, and Σ*_XX_*, Σ*_XY_*, and Σ*_YY_*, which is impossible for *p* > *n* and highly unstable when *p* is a large fraction of *n*. Besides this stronger filtering, all preprocessing steps remain the same as in section Example.

The left panel of **Figure 2** provides the analog of CCA loadings. To be precise, let *X* ∈ ℝ^102×36^ be the matrix of body composition measurements and *Y* ∈ ℝ^102x66^ be the variance-stabilized microbial abundances. As before, write *u_k_* ∈ ℝ^36^, *v*_k_ ∈ ℝ^66^ for the *k* ^th^ canonical correlation directions. Text labels from column *j* of the body composition variables are displayed at position ${\left(u_{j1},u_{j2}\right)}_{j=1}^{36}$ and shaded points for the *j* ^th^ species at position ${\left(v_{j1},v_{j2}\right)}_{j=1}^{66}$.

As in the concatenated PCA, we find that the groups of variables occupy separate spaces. Our interpretation is that sequences further to the left are correlated with the body variables further to the left, which are all in some way variants of body mass. Note that age is negatively correlated with total fat mass, which is why it appears on the opposite end. Among the abundant species that remain, there is limited clustering according to taxonomic group, though the Bacteroideceae and Ruminoccocus do appear restricted to the bottom right and left, respectively.

In the right panel of **Figure 2**, we plot the corresponding scores. Note that in CCA, there are two sets of scores for each *k*, the *Xu_k_* and *Yv_k_*. Indeed, the CCA objective finds directions that maximize the correlation between these scores. We use a different color legend for the two panels, each of which represents one set of scores. The legend for scores from species abundances are colored by family, while those for the body composition associates samples with android fat mass. The pairs of scores for each individual sample are drawn with small links. Since most links are relatively short, linear combinations of the two tables could be found that optimized the objective—indeed, the top two canonical correlations are 0.968 and 0.957. However, some caution is necessary here, and a more honest evaluation would be based on scores obtained by projecting new samples onto the original CCA directions. This is especially important in this nearly high-dimensional setting, where covariance estimation may be unreliable.

Aside from the fact that samples appear as pairs, interpretation proceeds as in a PCA scores plot, as in **Figure 1**. The association between these variables and the sample positions is not as strong as when performing PCA on the combined table. This is to be expected, however, as PCA maximizes variance without any thought to covariance, and the body composition table alone has a large portion of its variance related to android fat mass.

### Co-Inertia Analysis

Co-inertia Analysis (CoIA) emerged in ecology to facilitate analysis of variation in species abundance as a function of environmental conditions (Dolédec and Chessel, 1994). It can be viewed as a slight modification of CCA. Again, we seek sets of orthonormal directions ${\left(u_{k}\right)}_{k=1}^{K}$ and ${\left(v_{k}\right)}_{k=1}^{K}$ such that the associated projections *Xu_k_* and *Yv_k_* explain most of the covariation between the tables. Unlike CCA, CoIA finds its first directions by maximizing the covariance—not the correlation—between scores,

$$\begin{array}{l} \underset{u\in {\mathbb{R}}^{p_{1}},v\in {\mathbb{R}}^{p_{2}}}{\text{maximize}}\;u^{T}X^{T}\mathrm{Yv}\\ \mathrm{such}\;\mathrm{that}∥u∥\;=1\\ \;∥v∥\;=1, \end{array}$$

with subsequent directions found by the same optimization, after adding the constraint that they are orthogonal to the previously derived directions.

The only difference with the objective in equation (2) is that norm constraint is imposed on *u* and *v* directly, rather than their transformations $\sum_{\mathrm{XX}}^{\frac{1}{2}}u$ and $\sum_{\mathrm{YY}}^{\frac{1}{2}}v$. It is in this sense that the CCA objective maximizes the correlation between scores, while CoIA maximizes the covariance.

The solution ${\left(u_{k}\right)}_{k=1}^{K}$ and ${\left(v_{k}\right)}_{k=1}^{K}$ can be obtained as the first *K* left and right eigenvectors from the SVD of *X^T^Y*, as opposed to the first *K* generalized eigenvectors, as in CCA. The proof of this fact is almost identical to the derivation in section CCA, for CCA.

#### Example

**Figure 3:**
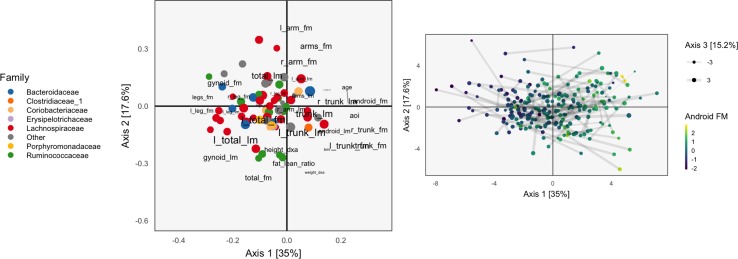
The Co-inertia Analysis (CoIA) analog of the PCA and CCA biplots in **Figures 1** and **2**. There seems to be a clearer gradient across android fat mass variables, though the scores are not so well aligned, since the links are somewhat longer.

We apply CoIA to the same data as used in section Example, as CoIA also needs to estimate the covariance between tables, which is difficult when the number of species is large. We find that the associated scores are quite different from those found using CCA. Compare **Figure 3**, which shades samples by android fat mass with **Figure 2** for CCA. The scores for CoIA are not so closely aligned across tables, but they exhibit a clearer gradient across android fat mass. We find that the scores are not nearly as closely aligned as they are for CCA, but that they are more strongly associated with variation in android fat mass, as in the concatenated PCA result of **Figure 1**. It is not clear whether this phenomenon—the CoIA scores being more similar to those from PCA than CCA—holds in general, or what it is about the change in inner products between CoIA and CCA that is responsible for this difference.

### MFA

MFA gives an alternative approach to producing scores and relating features across multiple tables (Pagés, 2014). It can be understood as a refined version of the concatenated PCA described in section PCA that reweights tables in a way that prevents any one table from dominating the resulting ordination. Specifically, MFA is a concatenated PCA on the matrix

$$X:=\left[\frac{1}{\lambda_{1}(X^{\left(1\right)})}X^{\left(1\right)}|\ldots |\frac{1}{\lambda_{1}(X^{\left(L\right)})}X^{\left(L\right)}\right],$$

which reweights each table *X* ^(^ *^k^* ^)^ by its largest eigenvalue, λ(*X*(*k*)). This procedure is the multitable analog of the common practice of standardizing variables before performing PCA.

The resulting MFA directions and scores can be interpreted in the same way as those from PCA—the MFA directions still specify the relationship between measured features, and the position of each sample’s projection describes the relative value of each feature for that sample. Moreover, MFA gives a way of comparing entire tables to each other, called a “canonical analysis” (Pagés et al., 2004). A *K*-dimensional representation of the *l* ^th^ group is given by

$$\left[ℒ\left(z_{1},X^{\left(l\right)}\right),\ldots ,ℒ\left(z_{K},X^{\left(l\right)}\right)\right],$$

where *z_k_* = *d_k_u_k_* ∈ ℝ*^n^* is the *k* ^th^ column of principal component scores and

$$ℒ\left(z_{k},X^{\left(l\right)}\right)=\frac{\lambda_{k}(X)}{\lambda_{1}(X^{\left(l\right)})}\text{tr}\left(X^{\left(l\right)}X^{(l)T}z_{k}z_{k}^{T}\right)=\frac{\lambda_{k}(X)}{\lambda_{1}(X^{\left(l\right)})}∥X^{(l)T}\mathrm{zk}{∥}_{2}^{2}$$

is a measure of aggregate similarity between the coordinates in the *l* ^th^ table and the *k* ^th^ column of scores. According to this definition, if the samples, as represented by the *l* ^th^ table, have high correlation with the *k* ^th^ dimension of scores, then the canonical analysis displays positions the *l* ^th^ table far in the *k* ^th^ direction. Plotting these table-level coordinates helps resolve which tables measure similar underlying variation.

### PCA-IV

PCA-IV adapts the dimensionality reduction ideas of PCA to the multivariate regression setting (Rao, 1964). It can also be viewed as a version of PCA that chooses a dimension reduction of *X* based on its ability to predict *Y*. In this sense, it anticipates methods like Partial Least Squares, Canonical Correspondence Analysis, the Curds & Whey procedure, and the Graph-Fused Lasso, which are described in sections Partial Least Squares, CCpnA, Curds & Whey, and Graph-Fused Lasso.

Formally, suppose we are predicting $y_{i}\in \;{\mathbb{R}}^{p_{1}}$ from $x_{i}\in \;{\mathbb{R}}^{p_{2}}$. . Since *p*_2_ may be large, it might be useful to work with a lower-dimensional representation $z_{i}=V^{T}x_{i}\in \;{\mathbb{R}}^{K}$. which is potentially more interpretable but still as (or more) predictive of *y_i_*. As in PCA, we require that *V* be orthonormal.

The criterion that PCA-IV uses to identify the loadings *V* and scores *Z* mirrors the maximum variance criterion for PCA. Instead of choosing *V* to maximize the variance of the *z_i_*, we choose it to minimize the residual covariance of *y_i_* given *z_i_*. That is, suppose that *y_1_* and *x*_1_ are jointly normal with mean 0 and covariance

$${\text{Var}}_{\mathbb{P}}\left(\begin{array}{c} y_{i}\\ x_{i} \end{array}\right)=\left(\begin{array}{ll} \Sigma_{\mathrm{YY}} & \Sigma_{\mathrm{YX}}\\ \Sigma_{\mathrm{XY}} & \Sigma_{\mathrm{XX}} \end{array}\right).$$

If *z_i_* = *VTx_i_*, then the joint covariance of *y_i_* and *z_i_* is

$${\text{Var}}_{\mathbb{P}}\left(\begin{array}{c} y_{i}\\ z_{i} \end{array}\right)=\left(\begin{array}{ll} \Sigma_{\mathrm{YY}} & \Sigma_{\mathrm{YX}}V\\ V^{T}\Sigma_{\mathrm{XY}} & V^{Τ}\Sigma_{\mathrm{XX}}V \end{array}\right),$$

so the residual covariance of *y*_1_ given *z*_1_ is

(4) $$\Sigma_{\mathrm{YY}}-\Sigma_{\mathrm{YX}}V{\left(V^{Τ}\Sigma_{\mathrm{XX}}V\right)}^{-1}V^{T}\Sigma_{\mathrm{XY}}.$$

Rao (Rao, 1964) uses the trace to measure the “size” of this matrix. The true population covariances are unknown to us, so we replace them by their empirical estimates. The formal optimization for PCA-IV then becomes

(5) $$\underset{V\in {\mathbb{R}}^{p_{2}\times K}\text{orthonormal}}{\text{minimize}}\text{tr}\left({\hat{\Sigma }}_{\mathrm{YY}}-{\hat{\Sigma }}_{\mathrm{YX}}V{\left(V^{T}{\hat{\Sigma }}_{\mathrm{XX}}V\right)}^{-1}V^{T}{\hat{\Sigma }}_{\mathrm{XY}}\right)$$

The optimal *V* are the top *K* generalized eigenvectors of ${\hat{\Sigma }}_{\mathrm{XY}}{\hat{\Sigma }}_{\mathrm{YX}}$ with respect to ${\hat{\Sigma }}_{\mathrm{XX}}$, that is, the orthonormal set of (*v_k_*) satisfying

$${\hat{\Sigma }}_{\mathrm{XY}}{\hat{\Sigma }}_{\mathrm{YX}}V=\left(\lambda_{1}{\hat{\Sigma }}_{\mathrm{XX}}v_{1}|\ldots |\lambda_{k}{\hat{\Sigma }}_{\mathrm{XX}}v_{k}\right)={\hat{\Sigma }}_{\mathrm{XX}}V\Lambda ,$$

where Λ = diag (*λ_k_*) ∈ ℝ*^K×K^*. A derivation for why this choice is optimal is provided in section *Derivation Details for PCA-IV*.

**Figure 4:**
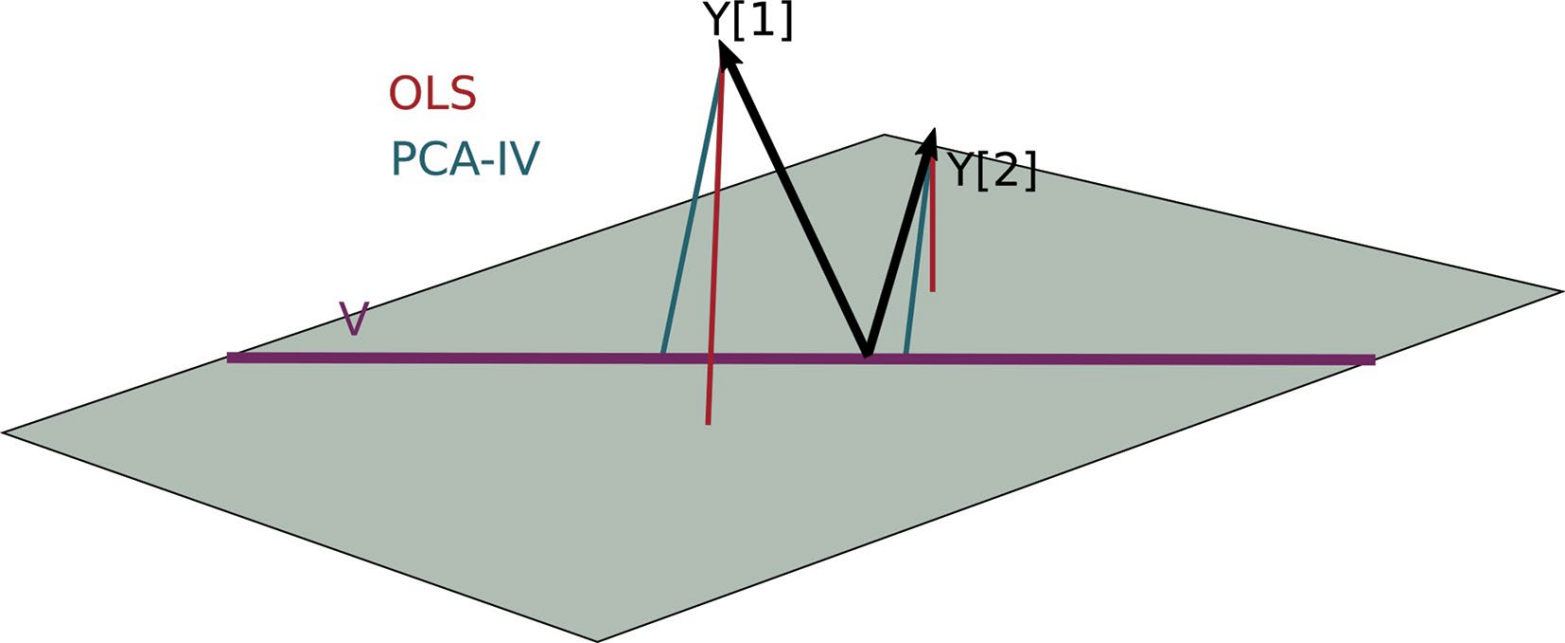
A geometric view of Principal Component Analysis with Instrumental Variables (PCA-IV). The columns of the response *Y* are views as *n*-dimensional vectors. The gray plane is the span of *X*. Multivariate OLS simply projects the columns of *Y* onto the plane, while PCA-IV searches for a further subspace *V* on which to project all responses.

For a geometric interpretation of PCA-IV, view each column *y_j_* in *Y* and *x_j_* in *X* as a point in ℝ*^n^*. Assuming *X* and *Y* are full rank, the collections (*y_j_*) and (*x_j_*) span *p*_1_- and *p*_2_-dimensional subspaces. A set of independent regressions of *y_j_* on *X* projects each individual *y_j_* onto the span of the (*x_j_*), and the squared residuals are the distance to this subspace. The PCA-IV procedure is an attempt to find a further *K*-dimensional subspace within the span of the (*x_j_*) such that the residuals of the regressions from *y_j_* onto this further subspace is not much worse. This is displayed in **Figure 4**.

#### Example

Continuing our WELL-China case study, we now illustrate results from PCA-IV. The idea of scores and loadings in this context requires some clarification. By PCA-IV scores, we mean the coordinates of projections *z_i_* of samples onto the subspace defined by *V*, and by loadings, we mean the correlation between columns^2^ of *X* and *Y* with the PCA-IV axes defining *V*.

**Figure 5:**
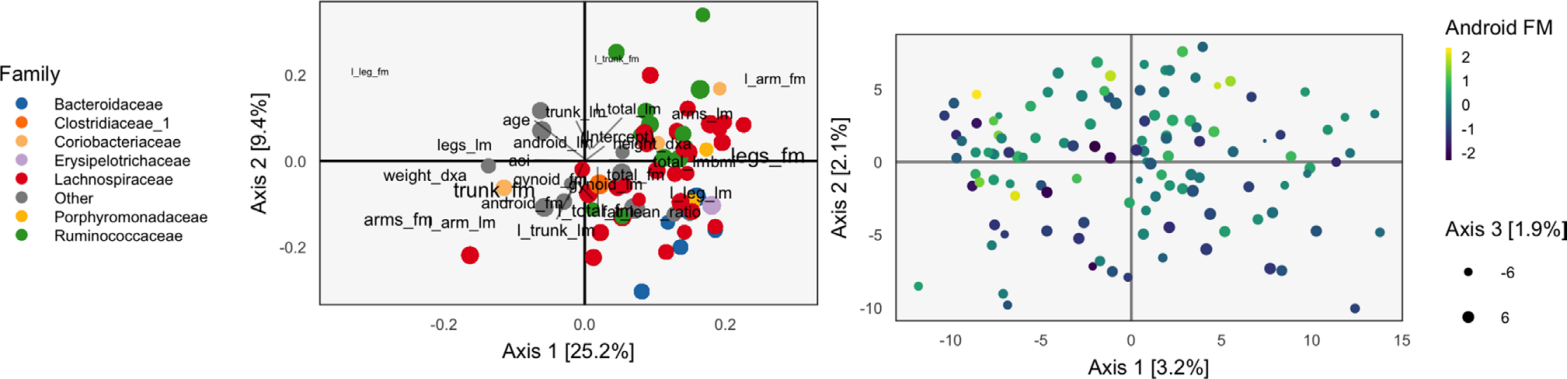
The PCA-IV biplot can be interpreted like biplots from previous methods, for example, **Figure 1**. Some of the relationships between variables seem less intuitive than those observed previously.

The scores and loadings are given in **Figure 5**. Interpretation of the species loadings is simple, since species seem well separated by taxa. Interpretation of the body composition variables is less clear—pairs of variables that would be expected to be near to one another are not, in many cases. Indeed, leg fat mass (leg_fm) and left leg fat mass (l_leg_fm) should have a small angle between one another, but they do not. It is possible that by approximating the covariation across tables, the quality of within-table approximations deteriorates.

We find that the scores, displayed in figures, are similar to those that found by the concatenated PCA of section PCA. One possible explanation for this behavior is that the PCA-IV-generalized SVD of *X* is similar to an ordinary PCA of *X*, and that in the concatenated PCA of (*Y X*), the fact that *X* has many more columns than *Y* means that the result is similar to a PCA on *X* alone.

### Partial Triadic Analysis

Partial Triadic Analysis (PTA) gives an approach for working with multitable data when each table has the same dimension, *p*1 = *p*2 (Kroonenberg, 2008; Thioulouse, 2011). Specifically, it gives a way of analyzing data of the form ${\left(X.._{l}\right)}_{l=1}^{L}$, where each *X*..*_l_* ∈ ℝ*^n×p^*. This is called a data cube because it can also be written as a three-dimensional array *X* ∈ ℝ*^n^* ^×^ *^p^* ^×^ *^L^*. We denote the *j* ^th^ feature measured on the *i* ^th^ sample in the *l* ^th^ table by *x_iji_*, and the slices over fixed *i*, *j*, and *l* by *X_i_*.., *X*.*_j_.*, and *X*..*_l_*. This type of data arises frequently in longitudinal data analysis, where the same features are collected for the same samples over a series of *L* times. However, the actual ordering of the *L* tables is not ever used by this method: if we scrambled the time ordering for *L* tables, the algorithm’s result would not change.

The main idea in PTA is to divide the analysis into two steps:

- Combine the *L* tables into a single compromise or consensus table.
- Apply any standard single-table method, e.g., PCA, on the compromise table.

A naive approach to constructing the compromise table would be to average each entry across the *L* tables. Instead, PTA upweights tables that are more similar to the average table, as these are considered more representative. Formally, the compromise is defined as $X_{c}=\Sigma_{l=1}^{L}\;\alpha_{l}X.._{l}=X\alpha \in {\mathbb{R}}^{n\times p}$, where *α* (constrained to norm one) is chosen to maximize $\sum_{l=1}^{L}\alpha_{l}\langle \bar{X},X.._{l}\rangle$, a weighted average of inner-products^3^ between each of the *L* tables and the naive-average table, $\bar{X}=\frac{1}{L}\sum_{l=1}^{L}X.._{l}$.

The optimal α can be derived using Lagrange multipliers (see *Derivation of PTA α*) and leads to the compromise table,

$$X_{c}=\sum_{l=1}^{L}\frac{\langle \bar{X},X.._{l}\rangle }{\sqrt{\sum_{l^{\prime }=1}^{L}{\langle \bar{X},X.._{l^{\prime }}\rangle }^{2}}}X.._{l}.$$

We can try to interpret the compromise matrix geometrically. Suppose the *X.._l_* define an orthonormal basis, so that $\langle X^{l},X^{l^{\prime }}\rangle =I(l=l^{\prime })$. Then, we can write the compromise table as

$$X_{c}=\sqrt{L}\sum_{l=1}^{L}\langle \bar{X},X.._{l}\rangle X.._{l}=\sqrt{L}\bar{X},$$

a scaled version of the mean.

If, however, the tables are not orthonormal, then we place more weight on directions that are correlated. For example, if *X* ^(1)^ = *X* ^(2)^, but the rest of the tables are orthogonal to each other and to these first two tables, then the compromise double counts the direction *X* ^(1)^. Therefore, compared to the naive average $\bar{X}$, *X_c_* upweights more highly represented tables.

### Statico and Costatis

In the multivariate ecology literature, it is common to have a pair of data cubes, giving species abundances and environmental variables over time, respectively. We write these as $Y\in {\mathbb{R}}^{n\times p_{1}\times L}$ and $Y\in {\mathbb{R}}^{n\times p_{2}\times L}$. Costatis and Statico are two approaches for analyzing such data (Thioulouse, 2011). They are easiest to understand as divide-and-conquer approaches, where the general problem of analyzing a pair of data cubes is divided into two steps, one designed for analyzing individual cubes, and another for studying covariation across tables. In Statico, the covariation problem is dealt with first, then followed by a data cube analysis, while in Costatis, that order is reversed.

Specifically, in Statico, an empirical cross-covariance matrix is constructed at each time point, $Z^{l}=\frac{1}{n_{l}}Y_{.._{l}}^{T}X_{.._{l}}$. For example, this is the correlation between the environmental variables and species counts at a specific time point *l*. The *L* matrices *Z^l^* are then input into a PTA, yielding a compromise table *Z_c_* that can then be studied with PCA.

Alternatively, in Costatis, a compromise table is constructed for each of the data cubes *Y* and *X*, using PTA. Call these *Y_c_* and *X*_c_. These are now simply two matrices, each with *n* rows, and they can be analyzed by any two-table dimensionality reduction method, for example, CoIA.

Hence, we see that the only difference between these methods is the order in which CoIA and PTA are applied. Indeed, this is reflected in the names of the methods: Statis is an abbreviation for a PTA, and Statico performs a CoIA before a Statis while Costatis does the reverse.

## Modern Multivariate Methods

Compared to classical approaches, modern multivariate methods are typically designed for more high-dimensional, heterogeneous settings. The two methods reviewed in this section are examples of this trend: Partial Least Squares (PLS) is well-suited for finding predictors in the presence of high-dimensional response matrices, while Canonical Correspondence Analysis (CCpnA) was designed to facilitate joint analysis of heterogeneous continuous and count data necessary. Unlike traditional statistical methods, neither approach is explicitly model-based, and both are iterative, requiring more extensive computation than earlier techniques.

### Partial Least Squares

PLS sequentially derives a set of mutually orthogonal features ${\left(z_{k}\right)}_{k=1}^{K}$ that characterizes the relationship between two tables, *Y* and *X* (Wold, 1985). To obtain the first PLS direction, *z*_1_, compute the first left singular vector *u*_1_ of the cross-covariance matrix between the two tables, ${\hat{\Sigma }}_{\mathrm{YX}}=\frac{1}{n}Y^{T}X$. Then, for each of the *p*_2_ columns of *X*, compute the univariate (i.e., partial) regression coefficient ${\hat{\varphi }}_{j}=\frac{1}{∥x._{j}{∥}_{2}^{2}}x_{._{j}}^{T}u_{1}$, for *j* = 1,…, *p*_1_. The first PLS direction is defined as $z_{1}=\sum_{j=1}^{p_{2}}{\hat{\varphi }}_{j}x._{j}$ a weighted average of *x*.*_j_* according to their partial correlation with *u*_1_. To generate subsequent directions *z_k_*, orthogonalize both *Y* and *X* with respect to the current directions *z*_1_,… *z_k_*_–1_, and repeat the process.

This procedure is appealing because, like PCA, it reduces a potentially high-dimensional matrix *X* with many correlated columns into a smaller set of orthogonal directions. Moreover, it achieves this reduction in a way that accounts for correlation with columns in *Y*: columns of *X* that are uncorrelated with *Y* will have no contribution to the PLS directions, even if they account for a large proportion of variation in *X*.

We have stated the procedure in the form it was originally proposed, but this algorithmic description is difficult to understand geometrically or probabilistically. However, interpretational aids have since been developed. Frank and Friedman (1993) and Stone and Brooks (1990) studied the case where *p*_1_ = 1, so *y* is a single column vector. By assuming that the rows of *y* and *X* are drawn i.i.d. from distribution ℙ*^YX^*, with marginals ℙ*^Y^* and ℙ*^x^*, they found that the *k* ^th^ PLS direction *z_k_* is the *z* that solves the optimization

(6) $$\begin{array}{l} \underset{z}{\text{maximize }}{\text{Corr}}_{{\mathbb{P}}^{\mathrm{YX}}}\left[x_{i}^{T}z_{k},y_{i}\right]{\text{Var}}_{{\mathbb{P}}^{X}}(z^{T}x_{i})\\ \mathrm{such}\text{ that }z^{T}X^{T}Xz_{j}=0\text{ for all j}\leq k-1\\ \;∥z{∥}_{2}=1. \end{array}$$

If the covariance term is omitted, the optimization is identical to the maximum variance problem that gives the principal component directions based on *X*. This formulation makes precise the idea that PLS is a version of principal components that accounts for correlation with *Y*.

An alternative interpretation, due to (Gustafsson, 2001), is that PLS fits a particular latent variable model. Suppose $\xi_{i}=(\xi_{i}^{s},\xi_{i}^{X})$ are drawn i.i.d. from a *K_1_ + K_2_ = K* dimensional spherical normal. PLS assumes the observed tables *Y* and *X* have rows drawn i.i.d. from

$$\begin{array}{l} y_{i}|\xi_{i}∼N(\mu_{Y}+W_{Y}\xi_{i}^{s},\sigma^{2}I_{p_{1}})\\ x_{i}|\xi_{i}∼N(\mu_{X}+W_{X}\xi_{i}^{s}+B_{X}\xi_{i}^{X},\sigma^{2}I_{p_{2}}). \end{array}$$

That is, each table is the sum of two components, one that is a table-specific linear combination of a shared latent variable, and another that is an arbitrary linear combination of a table-specific latent variable. The shared feature *ξ^s^* is the object of interest, and is what PLS implicitly estimates.

### Sparse Partial Least Squares

PLS suffers from two of the same problems as PCA:

- It can be unstable in high-dimensional settings, since it requires estimation of covariances, and isn’t well defined when *p* > *n*.
- PLS directions are linear combinations of all features in *x_i_*, which can be difficult to interpret when there are many features.

Different regularized, sparse modifications of PCA have been proposed to remedy these issues in the PCA context (Jolliffe et al., 2003; Zou et al., 2006; Witten et al., 2009). For PLS, similar analysis leads to sparse PLS (Lê Cao et al., 2008; Chun and Kele, 2010), and we briefly review this method here.

Directly regularizing the multiresponse version of the PLS optimization (6) leads to the problem

$$\begin{array}{l} \underset{z_{k}}{\text{maximize}}\sum_{j=1}^{p_{1}}{\text{Cov}}_{{\mathbb{P}}^{\mathrm{YX}}}\left[x_{i}^{T}z_{k},y_{\mathrm{ij}}\right]\\ \text{such that }z^{T}x^{T}xz_{j}=0\text{ for all }j\leq k-1\\ \;∥z_{k}{∥}_{\;2}=1\\ \;∥z_{k}{∥}_{\;1}\leq \lambda , \end{array}$$

which can be applied to real data by replacing the objective with its sample version, $z_{k}^{T}Mz_{k}$, where *M* = *X^T^YY^T^X*. This version of the problem falls into the Penalized Matrix Decomposition framework of Witten et al. (2009), reviewed in the section penalized matrix decomposition.

However, Chun and Kele (2010) argue that this formulation does not lead to “sparse enough” solutions. Instead, they adapt the SPCA approach of Zou et al. (2006) to PLS. The resulting objective identifies two sets of directions, a set (*a_k_*) that maximizes the PLS-defining covariance and another, (*z_k_*), that approximates the first set by a sparser alternative. Formally,

(7) $$\begin{array}{c} \underset{z_{k},a_{k}}{\text{maximize}}-\kappa ∥a_{k}{∥}_{\;M}^{\;2}+\;(1-\kappa )∥z_{k}-a_{k}{∥}_{M}^{2}\\ \text{such that }∥a_{k}{∥}_{\;2}^{\;2}=1\\ ∥z_{k}{∥}_{\;1}\leq \lambda_{1}\\ ∥z_{k}{∥}_{\;2}\leq \lambda_{2}, \end{array}$$

where we have defined $∥x{∥}_{M}=\sqrt{x^{T}\mathrm{Mx}}$ and *κ*, *λ*_1_, and *λ*_2_ are tuning parameters. The first term in the objective is the PLS-defining covariance, the second ensures that the solutions *z_k_* and *a_k_* are similar, and the norm constraints induce sparsity and stability on *z_k_*. Note that while this objective is not convex, for fixed *a_k_*, it is an elastic-net regression, while for fixed *z_k_*, it is a type of eigenvalue problem.

#### Example

Next we apply the sparse partial least squares (SPLS) implementation of Chung et al. (2012) to the WELL-China body composition data. We use the body composition variables as the response *Y* and the microbiome community composition as *X*. In this direction, a well-fitting model would allow the microbiome community measurements *X* to serve as a proxy for the variables in *Y*, in case those data were not easily accessible. To an extent, however, this choice of directionality is arbitrary—regressing abundances on body composition variables would also be sensible—and reflects the basic limitations of using an asymmetric method to study a symmetric problem.

**Figure 6:**
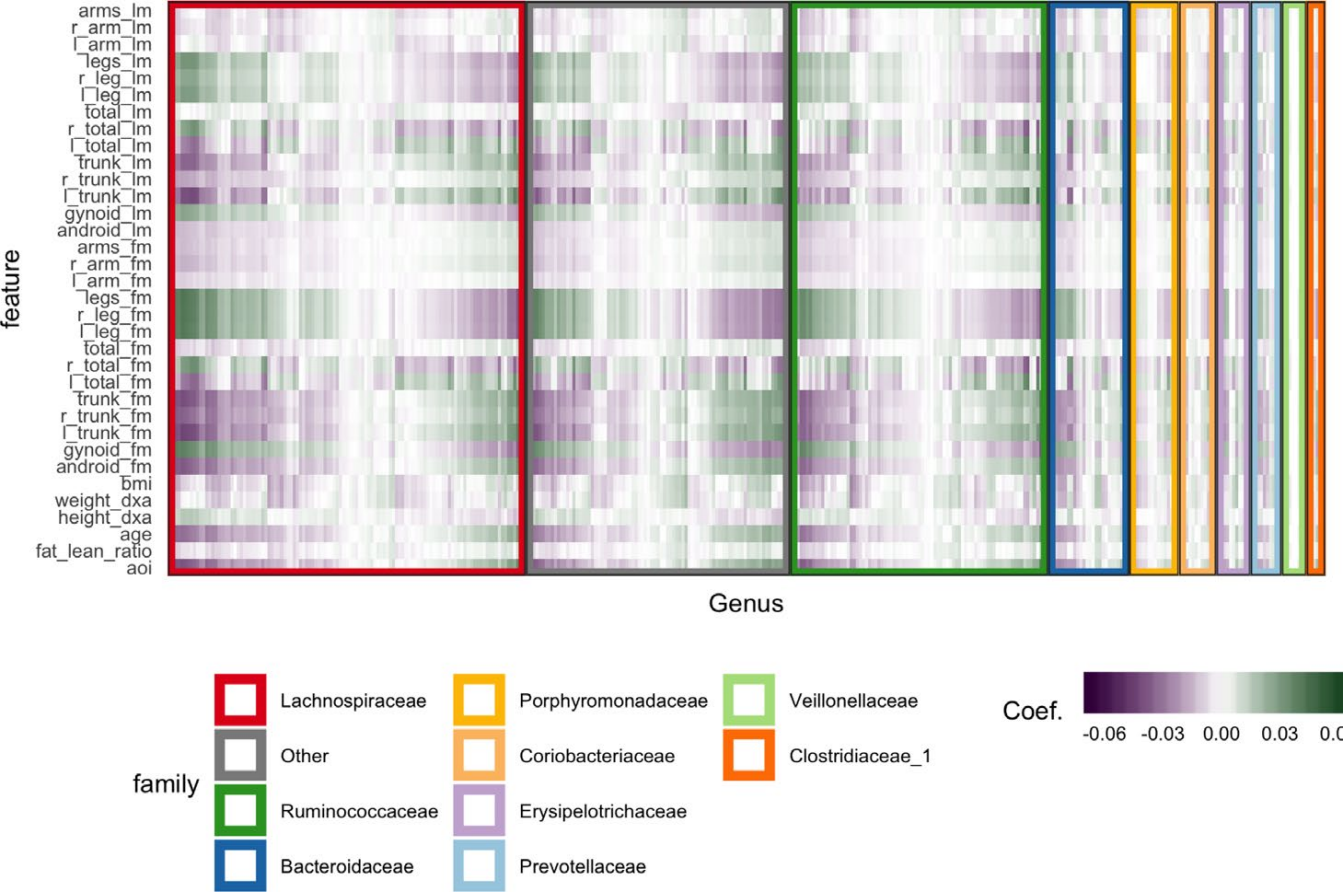
Coefficients learned by SPLS. Each row is a response dimension, which is a body composition variable. Each column is associated with a species. The shading within each cell corresponds to the SPLS coefficient for that species–response pair. Green and purple cells are positive and negative coefficients, respectively. Species are grouped first according to their taxonomic family, marked by grouping panel colors, and then by a hierarchical clustering on coefficient values.

We subset to female subjects and filter species, keeping only those species with a count of at least 5 in at least 7% of samples. This leaves 372 species over 119 participants. All species abundances are variance-stabilized using the approach of Anders and Huber (2010). We cross-validate with five folds, searching through a grid over *K* ∈ {4,…,8} and *λ*_1_∈ {0, 0.05,…, 0.7}. This grid is used to prevent the model from regularizing to the point that there is no information to visualize. For example, if we set *K* = 1, every row of **Figure 6** would look identical. The predictive accuracy is poor, which is unsurprising considering the spike at 0 in the abundances histogram—the held out error is ≈ 1.29, after having scaled and centered the body composition variables.

**Figure 6** displays fitted coefficients relating body composition variables with species abundances. By fitted coefficients, we mean we display $\hat{B}=ZQ^{T}$, where *Z* are the SPLS directions and a multiresponse linear regression model is used. Specifically, *Y* = *XB* + *E* = *XZQ^T^* + *E* where *X* is a matrix with rows *x_i_*, *Y* is a matrix with columns *y_j_*, and *Z* is a matrix with columns *z_k_*.

Positive associations tend to occur across all responses simultaneously, while negative associations can be unique to either lean or fat mass. Most taxonomic families seem to have slightly more negative than positive associations, with the possible exception of Porphyromonodaceae.

**Figure 7:**
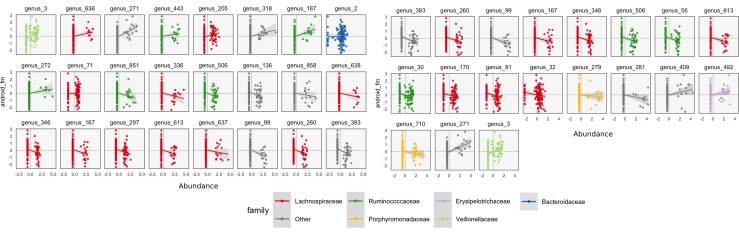
A more focused view of the species with high loadings according to SPLS (left) and sparse CCA (right). Each panel corresponds to a species. Points are shaded according to each species’ taxonomic family. The *x*-axis within panels corresponds to variance-stabilized species abundance, while the *y*-axis gives android fat mass. A linear smooth is provided to summarize the direction of associations. Panels are arranged according to the size of that species’ absolute SPLS coefficient value or loading onto the first sparse CCA axis. The presence of certain species seems to correspond to increased or decreased levels of android fat mass.

To interpret these coefficients in the raw data, we can visualize individual species with strong associations to body composition. Specifically, we study associations with the android and gynoid fat mass variables. In the left panel of **Figure 7**, we display the abundances *X* for species against android fat mass, respectively. The species are chosen according to whether the two-dimensional coefficient across android and gynoid fat mass has large norm^4^. The main associations that are visible are those between the body composition and species presence or absence. That is, there don’t seem to be any cases where a body composition feature varies smoothly as a species becomes more or less abundant. Instead, SPLS has identified species whose samples have lower or higher android or gynoid fat mass, depending on whether that species is present or absent.

### CCpnA

CCpnA is a method, originally developed in ecology, useful for joint analysis of count and continuous data. The canonical application has a site-by-species count matrix $Y\in {\mathbb{R}}^{n\times p_{1}}$ and an environmental features matrix $X\in {\mathbb{R}}^{n\times p_{2}}$, for example, historical rainfall and temperature measurements. In the WELL context, *Y* would be the samples by community abundance matrix, while *X* would contain the body composition measurements.

The scientific goal might be to identify species that are more abundant in sites with more rainfall or higher temperature. If these environmental variables were uncorrelated, it would be enough to fit a separate regression to each. This, however, is rarely the case, motivating the development for CCpnA.

Translating to the language of the WELL-study, individual samples can be thought of sites, and the supplemental data—that is, the body composition variables—are analogous to environmental variables.

CCpnA produces low-dimensional representations of both the rows and columns of *Y* (the samples and species), along with latent subspaces on which these representations are defined. Algorithmically, CCpnA first constructs the following matrices, where 1*_r_* denotes a column vector of *r* ones,

1. An overall frequency matrix,
  $$F=\frac{1}{n_{..}^{Y}}Y,$$
  where $n_{..}^{Y}$ is the sum of all counts in matrix *Y*.
2. A diagonal matrix of row (site) proportions,
  $$D_{r}=\text{diag(}F^{1}{}_{p_{1}}\text{)}\in {\mathbb{R}}^{n\times n}.$$
3. A diagonal matrix of column (species) proportions,
  $$D_{c}=\text{diag(}F^{T}{\text{1}}_{n}\text{)}\in {\mathbb{R}}^{p_{1}\times p_{1}}.$$
4. A projection onto the columns of the supplemental matrix *X*, reweighting samples according to their species counts,
  PX=Dr−12X(XTDrX)−1XTDr−12∈ℝn×n,

With this notation, compute an SVD,

$$D_{r}^{-\frac{1}{2}}=\left(F-F1_{p_{1}}1_{p_{1}}^{T}F\right)D_{C}^{-\frac{1}{2}}P_{X}=\mathrm{US}V^{T},$$

and define row and column scores *Z* and *Q* by

$$\begin{array}{l} Z=D_{r}^{-\frac{1}{2}}\mathrm{US}\\ Q=D_{c}^{-\frac{1}{2}}V^{T}S. \end{array}$$

There are several ways to interpret this procedure. CCpnA was originally proposed as the solution to a fixed-point iteration called reciprocal averaging (Ter Braak, 1986). Later, Greenacre (1984) and Greenacre and Hastie (1987), provided a geometric view and Zhu et al. (2005) gave an exact probabilistic interpretation.

The intuition for the reciprocal averaging procedure is simple: the scores for different samples should be a weighted average of the species scores, with larger weights for the species that are more common at those sites. Similarly, species scores can be defined according to a weighted average of sample scores. That is,

$$z_{i}\propto \frac{1}{f_{i}.}\sum_{j=1}^{P1}f_{\mathrm{ij}}q_{\mathrm{ij}}$$

$$q_{i}\propto \frac{1}{f_{.j}}\sum_{i=1}^{n}f_{\mathrm{ij}}z_{\mathrm{ij}},$$

or, in matrix form,

$$Z\propto \text{diag (}F1p_{1}{\text{)}}^{-1}FQ^{T}$$

$$Q\propto \text{diag (}F^{T}1_{n}{\text{)}}^{-1}Z.$$

This formulation suggests an algorithm for finding *Z* and *Q*—arbitrarily initialize one and iterate these calculations until convergence.

As is, this is not yet the setup that yields CCpnA—it does not use information in the supplemental table *X*. To recover CCpnA, a projection step needs to be inserted before the calculation of row scores,

1. Arbitrarily initialize *Z*.
2. While not converged,
  - $\text{Solve }Q^{\prime }\propto \text{diag (}F^{T}1_{n}{)}^{-1}F^{T}Z.$
  - $\text{Project }Q\text{ = }P_{X}Q^{\prime }\text{.}$
  - $\text{Solve }Z\propto \text{diag (}Z{\text{1}}_{p_{\text{1}}}{\text{)}}^{-1}FQ^{T}.$

The fixed point of this iteration is the previously described CCpnA solution.

A second interpretation is due to Zhu et al. (2005). Suppose first that we are only interested in a one-dimensional score for rows and columns. Let *α* be a latent gradient, for example, between warm-dry and cold-wet sites, or low and high android-fat mass samples. For each of the *p*_1_ species, define a normal density over the supplemental variables, $f_{j}(x_{i})=N(x_{i}|\mu_{j},\Sigma_{j})$. The mode of this density represents the preferred environment for species *j*. Next, project these densities onto the gradient, giving a univariate $f_{j}^{\alpha }(z_{i})=N(z_{i}|\alpha^{T}\mu_{j},\alpha^{T}\Sigma_{j}\alpha )$ for each species. The *z_i_* represent the scores for species *i* along the gradient *α*.

The generative model views species–sample pairs one at a time. For each pair involving sample *i* and species *j*, draw a score according to $f_{j}^{\alpha }(z_{i})$. Hence, each site *i* draws species according to a *p*_1_-class linear discriminant (LDA) model.

To use this idea to compute scores, we need to estimate the gradient α, which is also of interest in its own right. This is done by supposing equal covariances across species, Σ*_j_* = Σ for all *j*, and finding the $\hat{\alpha }$ maximizing the between vs. total variance across species,

$$\frac{\alpha^{T}\sum_{B}\alpha }{\alpha^{T}\sum \alpha },$$

where

$$\sum_{B}=\sum_{j=1}^{p_{1}}f._{j}(\mu_{j}-\bar{\mu }){\left(\mu_{j}-\bar{\mu }\right)}^{T}$$

is a between-species covariance matrix. Estimating $\hat{\alpha }$ in this way and writing $z_{i}={\hat{\alpha }}^{T}x_{i}$ gives the original site scores from CCpnA.

We have omitted a detailed numerical example of this method in this review, but note that codes for applying this method are available in the github repository associated with this review.

### Penalized Matrix Decomposition

In high-dimensional settings, sparsity is a desirable property, for both qualitative interpretability and statistical stability. A regression model using only a few features is easier to understand than one involving a linear combination of all possible features. Further, regularized models typically outperform their unregularized counterparts in terms of both predictive accuracy and inferential power (Buhlmann and Van De Geer, 2011). In fact, it is impossible to fit an unregularized linear regression when the number of features is greater than the number of samples.

The Penalized Matrix Decomposition (PMD) is a general approach to adapting the regularization machinery developed around regression to the multivariate analysis setting (Witten et al., 2009). The CCA and MultiCCA instances of PMD have been particularly well-studied (Witten et al., 2009; Witten et al., 2013).

The general setup is as follows. Suppose we want a one-dimensional representation of the samples (rows) in *X* ∈ ℝ^n×^ *^p^*. Recall that the first *k*-eigenvectors recovered by PCA span a subspace that minimizes the *ℓ* ^2^-distance from the original data to their projections onto that subspace. In particular, when *k* = 1, the associated PCA coordinates *u* ∈ ℝ*^n^* and eigenvector *v* are the optimal values in the problem

$$\begin{array}{l} \underset{u\in {\mathbb{R}}^{n},v\in {\mathbb{R}}^{p},d\in \mathbb{R}}{\text{minimize}}∥X-{\mathrm{duv}}^{T}{∥}_{2}^{2}\\ \text{ subject to }∥u{∥}_{2}^{2}=∥v{∥}_{2}^{2}=1. \end{array}$$

The PMD generalizes this formulation of rank-one PCA to enforce additional structure on *u* and *v*. The PMD solutions *u* and *v* are defined as the optimizers of

(8) $$\begin{array}{l} \underset{u\in {\mathbb{R}}^{n},v\in {\mathbb{R}}^{p},d\in \mathbb{R}}{\text{minimize}}∥X-\mathrm{du}v^{T}{∥}_{2}^{2}\\ \text{subject to }∥u{∥}_{2}^{2}=∥v{∥}_{2}^{2}=1.\\ \;{\text{Pen}}_{u}(u)\leq \mu_{1}\\ \;{\text{Pen}}_{v}(v)\leq \mu_{2} \end{array}$$

where Pen*_u_* and Pen*_v_* are arbitrary constraints *u* on and *v*.

To choose the regularization parameters *μ*_1_ and *μ*_2_, Witten et al. (2009) applied cross-validation to the reconstruction errors after holding out random entries in *X*. To obtain a sequence of scores ${\left(u_{k}\right)}_{k=1}^{K}$ and ${\left(v_{k}\right)}_{k=1}^{K}$ for *K* > 1, define *u_k_* and *v_k_* as the optimizers of the problem (equation 8) on the residual: $X^{k}:=X^{k-1}-d_{k-1}u_{k-1}v_{k-1}^{T}$ where $d_{k}=u_{k}^{T}X^{k}v_{k}$ and *X* ^1^ = *X*.

This view can be specialized to develop regularized versions of a number of multivariate analysis problems. We consider applications to the CCA and MultiCCA problems. Recalling that $∥A{∥}_{F}^{2}=\text{tr(}A^{T}\;A\text{)}$ along with the linearity and the cyclic properties of the trace, the objective in equation (8) can be rewritten, using ≡ to mean equality up to terms constant in *u* and *v*,

$$\begin{array}{l} ∥X-\mathrm{du}v^{T}{∥}_{F}^{2}=\text{tr}\left({\left(X-\mathrm{du}v^{T}\right)}^{T}\left(X-\mathrm{du}v^{T}\right)\right)\\ \;\equiv -2d\text{ tr}\left(X^{T}uv^{T}\right)+d^{2}\mathrm{tr}\left(uv^{T}uv^{T}\right)\\ \;\equiv -2dv^{T}X^{T}u+d^{2}, \end{array}$$

where for the last equivalence we used that *v^T^v* = *u^T^u* = 1.

From this expression, and by partially minimizing out *d* = *v* *^T^X^T^u*, we see that the PMD solutions *u* and *v* in equation (8) can be found as the optimizers of

$$\begin{array}{l} \underset{u\in {\mathbb{R}}^{n},v\in {\mathbb{R}}^{p}}{\text{maximize}}u^{T}X^{T}v\\ \text{subject to ||}u|{|}_{2}^{2}=\text{||}v|{|}_{2}^{2}=1\\ \;{\text{Pen}}_{u}(u)\leq \mu_{1}\\ \;{\text{Pen}}_{v}(v)\leq \mu_{2} \end{array}$$

Notice that, as long as the penalties are convex in *u* and *v*, the optimization is biconvex, so a local maximum can be found by alternately maximizing over *u* and *v*.

From this form, we can derive a sparsity-inducing version of CCA. Recall the maximal-covariance interpretation of CCA,

$$\begin{array}{l} \underset{u\in {\mathbb{R}}^{p_{1}},v\in {\mathbb{R}}^{p2}}{\text{maximize}}\;u^{T}{\hat{\sum }}_{\mathrm{XY}}v\\ \text{ subject to }u^{T}{\hat{\sum }}_{\mathrm{XX}}u=v^{T}{\hat{\sum }}_{\mathrm{YY}}v=1 \end{array}$$

Witten et al. (2009) argue for diagonalized CCA, in which the variance constraints are replaced by unit norm constraints, and sparsity-inducing *ℓ* ^1^ constraints are added,

$$\begin{array}{l} \underset{u\in {\mathbb{R}}^{p_{1}},u\in {\mathbb{R}}^{p2}}{\text{maximize}}u^{T}{\hat{\sum }}_{\mathrm{XY}}v\\ \text{subject to }||u{||}_{2}^{2}=\;||v{||}_{2}^{2}=1\\ \text{ ||}u{||}_{1}\leq \mu_{1}\\ \text{ ||}u{||}_{1}\leq \mu_{2} \end{array}$$

which is exactly of the form of equation (9) where $X={\hat{\sum }}_{\mathrm{XY}}$.

Multiple CCA can also be described in this framework, by replacing the objective with the sum over all pairwise covariances, $\sum_{l,l^{\prime }=1}^{L}c_{1}^{(l)T}X^{(l)T}X(l^{\prime })c_{1}^{(l{)}^{\prime }}$, and introducing constraints for each of the $c_{1}^{\left(l\right)}$.

#### Example

We apply the PMD formulation of sparse CCA to the WELL-China data. As before, we *k*-over-*A* filter the microbiome data, requiring species to have counts of at least 5 in at least 7% of samples. Further, we first variance-stabilize, center, and scale these species abundances. For the regularization parameters, we set *μ*_1_ = 0.7 for the body composition data and *μ*_2_ = 0.3 for the species count data. The reasoning behind the relative values of these two tuning parameters is that sparsity in species loadings is more important than sparsity across body composition variables, because the microbiome data are more high-dimensional. The choice of the tuning parameters’ overall magnitude was guided by the overall number of factors that we wanted to retain.

We only compute the first three PMD directions, and the associated correlations between scores are (*d*_1_, *d*_2_, *d*_3_) = (0.700, 0.435, 0.632). Note that the correlation can increase in subsequent directions, since directions are computed iteratively and cannot be defined and sorted all at once.

**Figure 8:**
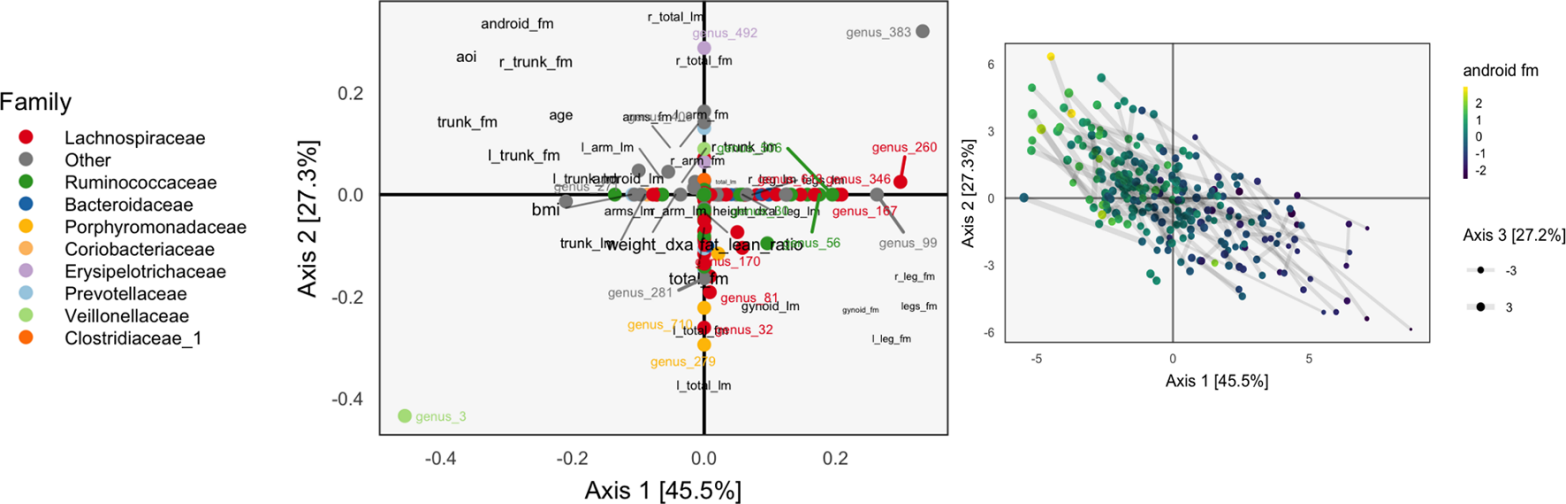
A sparse CCA biplot, for variables with at least one nonzero coordinate. In the loadings (left), each point corresponds to a species, and is shaded in by tax onomic family. Species with loadings far from the origin are also annotated with their names. Black text are loadings for body composition variables. The size of points and text reflects the contribution of the third CCA dimension. Many loadings have at least one dimension that is exactly zero, due to *ℓ* ^1^-regularization. For the sample scores (right), each point is a sample, positioned at their coordinates with respect to the first two learned sparse CCA directions. Points are shaded according to android fat mass, and their sizes are set according to the third sparse CCA direction’s contribution. Evidently, the first two directions reflect a gradient across android fat mass, suggesting that this is a substantial contributor to covariation across microbiome and body composition tables.

The learned loadings and scores are displayed in **Figure 8**. The *x*-axis in the loadings differentiates between high android and gynoid fat mass. The *y*-axes in the loadings reflect a gradient between overall right and left body mass. The size of points corresponds to the third PMD direction, and it seems to highlight high BMI, ratio of fat to lean mass, and overall weight. We interpret species based on their positions relative to these body composition variables, as in an ordinary biplot. For example, genus 492, located in the center-top, seems to be more common among people with higher android and lower gynoid fat mass.

The associated scores are displayed in the right panel, shaded according to android fat mass. The gradient between android and gynoid fat mass suggested by the loadings is clearly visible from this display. The length of links reflects the correlation between sets of scores. They are somewhat longer in the sparse CCA compared to the ordinary CCA on a subset of species, but this is likely a consequence of regularization and overfitting on the part of ordinary CCA.

We can follow up these displays by focusing on species that seemed related to the CCA axes. In the right panel of **Figure 7**, we isolate species with loa dings a distance of at least 0.15 from the origin. These are the same ones that are labeled by text in **Figure 8**. We can see associations between abundance and android fat mass, as suggested by the loadings. Generally, there is a difference between android fat mass among people with and without particular species—there is no smooth function between the quantity of a species android fat mass, even in these cases where an association exists. Further, no individual taxonomic group seems to dominate the set of associated species.

### Multitable Mixed-Membership

In section CCA, a latent variable interpretation of CCA was provided as an alternative to the standard covariance maximization perspective. Since likelihood-based methods are easily adapted to different data types, it is natural to consider versions of CCA designed for non-Gaussian data, using section CCA as a starting point. We are particularly interested in data with the same structure as the WELL-China body composition and microbiome data, namely, two table data where one table is continuous with Gaussian marginals and correlated columns and the other is a high-dimensional collection of counts, where many entries are exactly zero.

As before, define a set of shared scores $\xi_{i}^{s}\in {\mathbb{R}}^{K}$, and two sets of within-table scores $\xi_{i}^{X}\in {\mathbb{R}}^{L_{1}}$ and $\xi_{i}^{Y}\in {\mathbb{R}}^{L_{2}}$. As before, we model the body composition variables using essentially a Gaussian factor analysis model, $y_{i}|\xi_{i}^{X},\xi_{i}^{Y}∼N(B^{y}\xi_{i}^{s}+W^{y}\xi_{i}^{y},\sigma^{2}I_{p_{2}})$ with a spherical Gaussian prior $\xi_{i}^{X},\xi_{i}^{Y}$ on. For the counts matrix, we might consider a few different approaches:

- *Bayesian Exponential Family PCA* (Mohamed et al., 2009): By requiring low-rank structure on the natural parameters of an exponential family model, we could naturally model high-dimensional count data, using a Poisson or multinomial likelihood, for example.
- *Nonnegative Matrix Factorization* (Lee and Seung, 2001): A variant of the exponential family approach is to model the counts matrix as a Poisson likelihood over a low-rank product of Gamma random matrices.
- *Latent Dirichlet Allocation* (LDA) (Blei et al., 2003): We can model the observed samples as Dirichlet mixtures of a few underlying “topics,” which are themselves drawn from a Dirichlet prior.

Here, we focus on the LDA approach, though we suspect that the other two approaches are potentially interesting as well. Formally, this model supposes that counts are drawn according to

$$\begin{array}{c} x_{i}|(\theta_{k})∼\text{Mult}\left(x_{i}\text{|}N_{i}\sum_{k=1}^{K}\theta_{\mathrm{ik}}\beta_{k}\right)\\ \theta_{i}∼\text{Dir (}\alpha \text{)}\\ \beta_{k}∼\text{Dir (}\gamma \text{),} \end{array}$$

where $N_{i}=\sum_{j=1}^{p_{1}}x_{\mathrm{ij}}$ is the total count in sample *i*. This has the flavor of a factor analysis where ${\left(\theta_{\mathrm{ik}}\right)}_{k=1}^{K}$ are scores for the *i^th^* sample and (*β_k_*) are *K* underlying topics.

The only complexity with using an LDA model of *X* together with a Gaussian factor analysis on *Y* is that the shared scores $\xi_{i}^{s}$ typically have different priors—a Dirichlet for LDA and a spherical Gaussian for factor analysis. In any formulation of probabilistic CCA that uses both models, this must be reconciled. One approach is to continue to place Dirichlet priors on all the scores, $\xi_{i}^{s},\xi_{i}^{x}$, and $\xi_{i}^{y}$. While the model for the Gaussian data is no longer exactly traditional factor analysis, it has a similar interpretation. Alternatively, we could use a spherical Gaussian prior on all scores and then recover probability vectors by applying the softmax function, ${\left[S(v)\right]}_{k}=\frac{\exp(v_{k})}{\sum k^{\prime }\exp(v_{k^{\prime }})},$

$$\begin{array}{c} x_{i}\text{|}\xi_{i}^{s},\xi_{i}^{x}∼\text{Mult}\left(x_{i}\text{|}N_{i},S\left(B^{X}\xi_{i}^{s}+W^{X}\xi_{i}^{x}\right)\right)\\ \xi_{i}^{s}∼N(\xi_{i}^{s}\text{|}0,\tau^{2}). \end{array}$$

It is this second model that we use in our experiments below.

#### Example

We illustrate this multitable mixed-membership approach on the WELL-China data. We choose *K* = 2 for the number of shared topics and *L*_1_ = *L*_2_ = 3 for the number of unshared topics per table. We initialize scores and loadings using results from the PMD formulation of sparse CCA. While the use of shared $\xi_{i}^{s}$ and unshared $\left(\xi_{i}^{x},\xi_{i}^{y}\right)$ scores gives more flexibility in modeling, it also leads to additional complexity in interpretation—there are both more scores and more loadings that need to be visualized.

**Figure 9:**
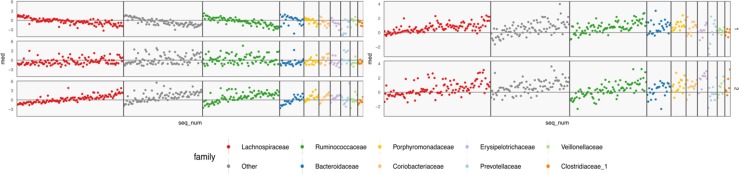
Table-specific (left) and cross-table (right) loadings for different species. Each row is a loading dimension, columns are features (species in this case), and intervals summarize posterior samples for the associated loading parameter, $W_{\mathrm{jk}}^{X}$ for table-specific loadings, and $B_{\mathrm{jk}}^{X}$ for cross-table loadings. Species are sorted from most to least abundant, within each taxonomic family. Caution must be exercised when interpreting these loadings, as loadings are invariant under rotations and reflections.

**Figure 10:**
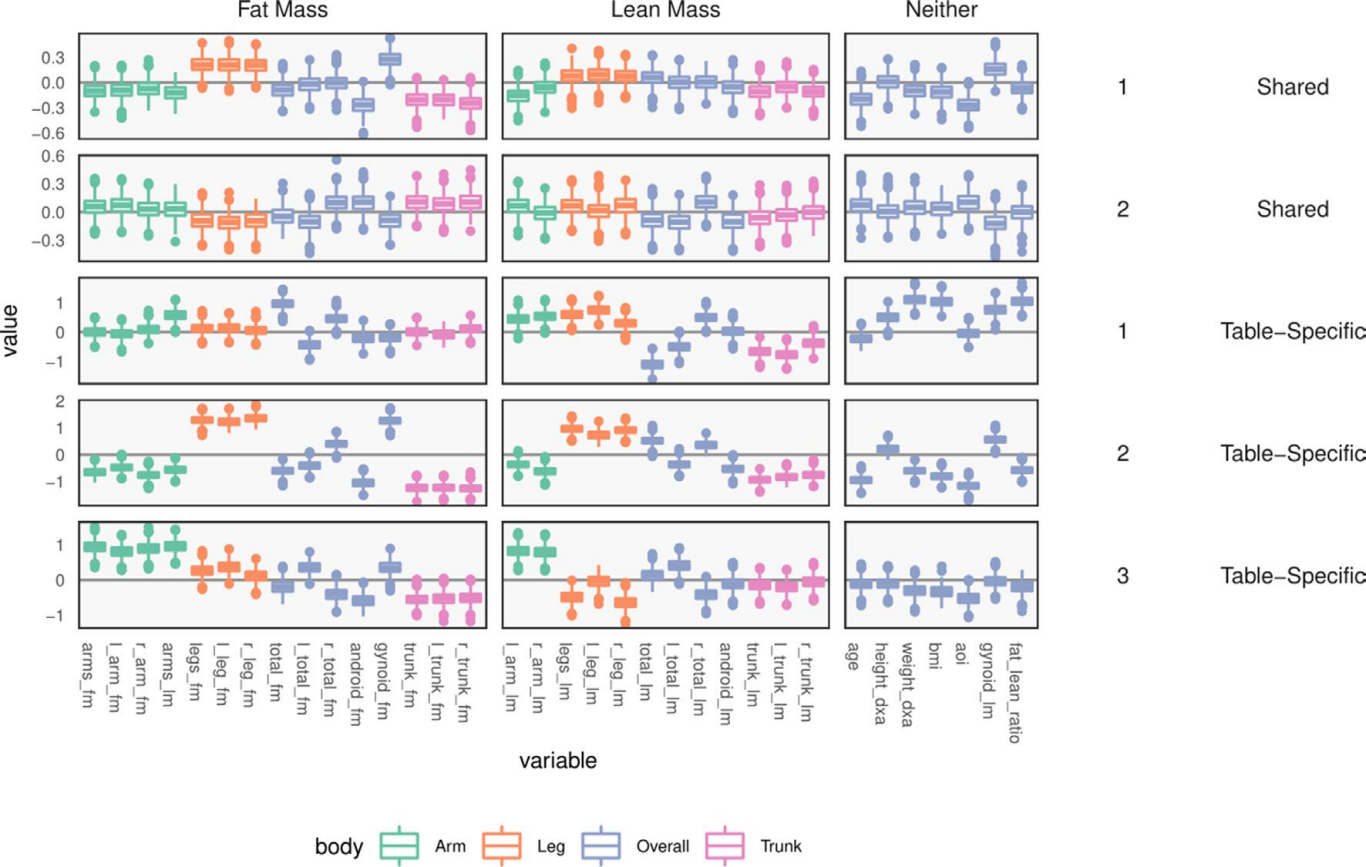
Table-specific and shared loadings, for the body composition variables, corresponding to the parameters $W_{\mathrm{jk}}^{Y}$ and $B_{\mathrm{jk}}^{Y}$ As in **Figure 9**, each row is one loading dimension, columns are features, and boxplots summarize posterior samples for the associated loading parameters. Colors distinguish between parts of the body. We note that loadings learn specific contrasts between types of fat mass and parts of the body.

Consider the loadings *W^X^* and *W^Y^*, provided in the left panel of **Figure 9** and bottom three rows of **Figure 10**. Note that there is no notion of variance explained by different axes in this case.

The loadings *W^X^* of **Figure 9** summarize table-specific variation in bacterial abundances. Invariance under rotation and reflection complicates interpretation of these estimates. If we flip the sign of all the loadings axes, then the more abundant species have larger loadings, so the direction of different trends is irrelevant. The main distinction between the first and second loadings is the rate of decay in frequencies, especially among Lachnospiraceae and Ruminococcaceae. For example, topic 1 seems to include species from these taxonomic families that are not very abundant. The main characteristic of the third loading is that it has higher values for Porphyromonadaceae, so samples with high weight on this loading have decreased levels of these taxa.

Next, consider within-table body composition loadings, given in the bottom three rows of **Figure 10**, which suggests that the first and third axes of *W^Y^* capture variation between overall and android vs. gynoid fat mass. The first axis has high loadings for weight, BMI, and total fat mass, and the third contrasts areas with high android and high gynoid fat mass. The second axis distinguishes between right and left total lean and fat mass variation, while the third axis captures difference between mass in the trunk versus arms and legs.

These summaries could have been obtained by analyzing each table separately. Covariation between the two tables is captured by the shared scores $\xi_{i}^{s}$ and loadings *B^X^*, *B^Y^*. The shared body composition loadings are given in the top two rows of **Figure 10**. These loadings again differentiate between android and gynoid fat mass, learning contrasts between body mass in arms and legs, for example, though the effects are less pronounced than in the table-specific loadings.

The shared bacterial abundance loadings are given in the right panel of **Figure 9**. The most notable observation is that the first axes places more weight on rarer species, while the second places proportionally more weight on abundant species. Further, the two axes seem to have very different behaviors with respect to Prevotellaceae and Veillonellaceae.

In general, we find the results from the LDA–CCA approach less satisfying than those of the sparse CCA of section Penalized Matrix Decomposition. It seems that inference of a probabilistic model with shared and unshared parameters is more difficult than optimization of a single set of shared parameters. It may be possible to improve this approach through the following strategies:

- Applying LDA–CCA only to those species that are not sent entirely to zero by sparse CCA.
- Placing a sparsity-inducing prior on the scores *B^X^*, *B^Y^*, *W^X^*, and *W^Y^*, respectively, in the spirit of Archambeau and Bach (2009).

### Curds & Whey

The Curds & Whey (C&W) procedure is a “soft” version of reduced-rank regression, differentially shrinking the ordinary least squares (OLS) fits with respect to the response canonical correlation directions (Breiman and Friedman, 1997). This is in contrast to reduced-rank regression, whose projection onto the first K response canonical correlation directions is a hard-thresholding analog. Hence, C&W is to reduced-rank regression what ridge regression is to principal component regression.

More precisely, the C&W algorithm fits a table *Y* according to

(10) $$\hat{Y}=P_{X}\mathrm{YV}\Lambda^{-1},$$

where again $V\in {\mathbb{R}}^{p_{1}\times p_{1}}$ are the CCA directions associated with the response *Y* and *P_x_* is the projection operator onto the column space of *X*. Λ is defined to be a diagonal matrix that determines the degree of shrinkage for the different canonical directions.

The main difficulty in C&W is the choice of Λ, and Breiman and Friedman (1997) suggest several possibilities. One choice is derived from a generalized cross-validation point of view, and results in shrinkage towards the response canonical correlation directions, without assuming the form of equation (10) *a priori*. This derivation is provided in section *Derivation of Curds & Whey Shrinkage*.

### Graph-Fused Lasso

An approach to multiresponse regression, introduced by Chen et al. (2010), incorporates prior knowledge about the relationship between responses. Specifically, they use the correlation network between responses to induce structured regularization on the regression parameters.

Let $Y\in {\mathbb{R}}^{n\times p_{1}}$ and $X\in {\mathbb{R}}^{n\times p_{2}}$ and assume a correlation network between the *p*_2_ tasks. This is denoted by *G* = (*V, E*), where *V* = {1,…,*p*_1_}. Each edge *e* is associated with a weight, *r* (*e*), giving the correlation between the pair of responses.

The graph-fused lasso estimates a coefficient matrix $B\in {\mathbb{R}}^{p_{2}\times p_{1}}$ whose columns *β* ^(^ *^r^* ^)^ are the regression coefficients across tasks, but which have been pooled together, with the strength of the pooling depending on the separately computed strength of the relationship between tasks. Formally, $\hat{\beta }$ is defined as the solution to the optimization,

(11) $$\underset{B\in {\mathbb{R}}^{p_{2}\times p_{1}}}{\text{minimize}}\frac{1}{2}\text{||}Y-\mathrm{XB}{\text{||}}_{F}^{2}+\lambda \text{||}B{\text{||}}_{1}+\gamma \sum_{e\in E}\sum_{j=1}^{p_{2}}\text{|}r_{e}\text{|}\left|\beta_{j}^{\left(e+\right)}-\text{sign(}r_{e}\text{)}\beta_{j}^{\left(e-\right)}\right|,$$

where ||*B*||_1_ is the sum of the absolute values of all entries of *B*, *β_j_* is the *j* ^th^ row of *B*, and *e* ^−^ and *e* ^+^ denote the nodes at either end of the edge *e*. The last regularization term in the objective is called the graph fused-lasso penalty, and it is this element that encourages pooling of information across regression problems.

### Example

We apply the graph-fused lasso to the body composition problem and compare it to a naive version of the lasso that does not share any information across responses. We consider predicting the body composition variables, many of which are strongly correlated with one another, using variance-stabilized bacterial abundances.

We filter away species that do not appear in at least 7% of samples, as in the original PCA approach. We set the smoothing parameter to *μ* = 0.01, while the *ℓ* ^1^ and graph-regularization parameters are set to *λ* = 0.1 and *γ* = 0.01, respectively, after they were heuristically found to provide interpretable levels of sparsity and smoothness in the fitted coefficients.

**Figure 11:**
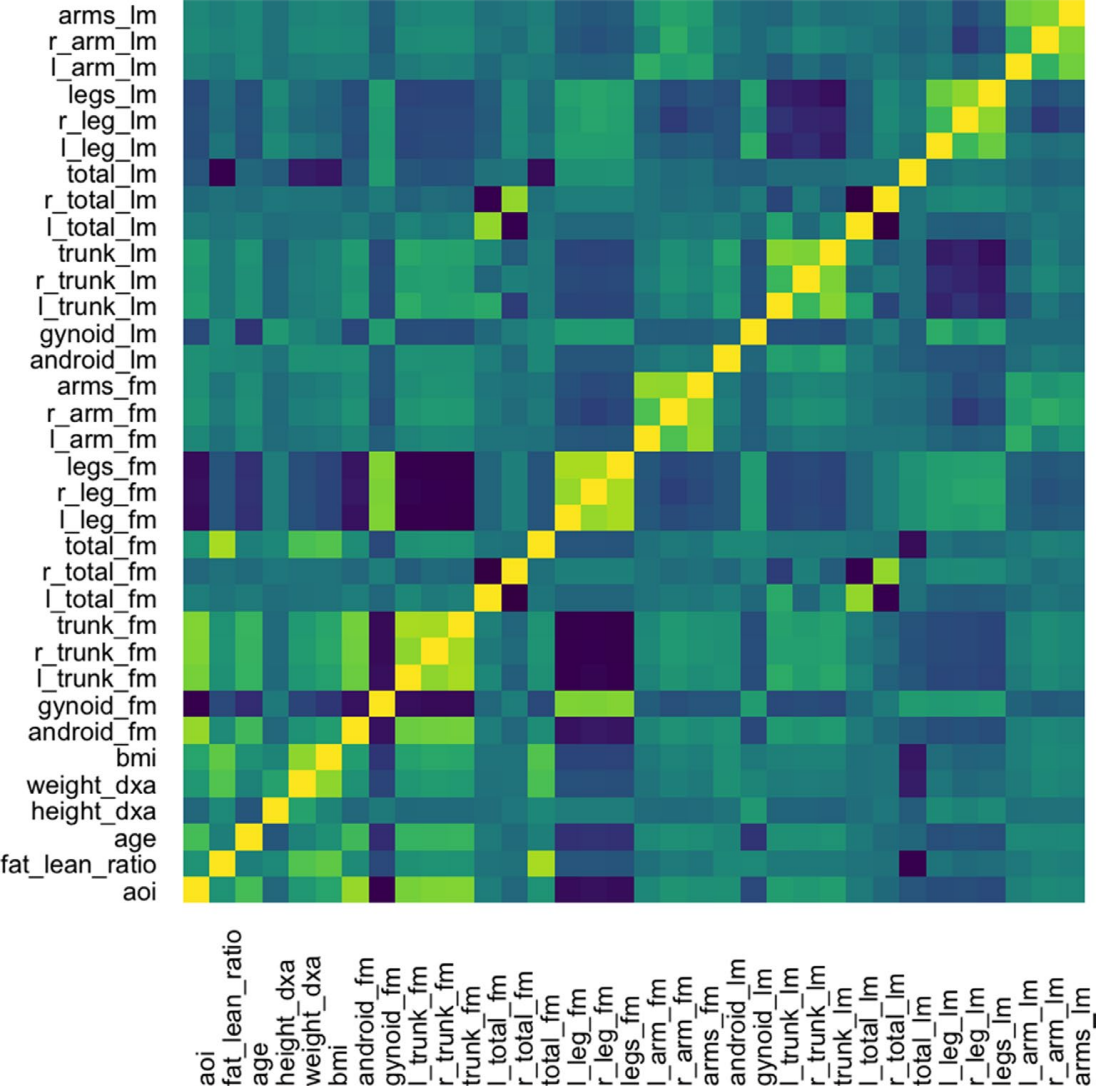
Correlation matrix used as the input graph *R* for the graph-fused lasso, estimated itself according to the graphical lasso.

The graph-fused lasso requires a correlation graph between response variables. We estimate such a graph using the graphical lasso (Friedman et al., 2008), since there are only ∼100 with which to estimate the 36-dimensional covariance matrix. The estimated correlation matrix is displayed in **Figure 11**.

**Figure 12:**
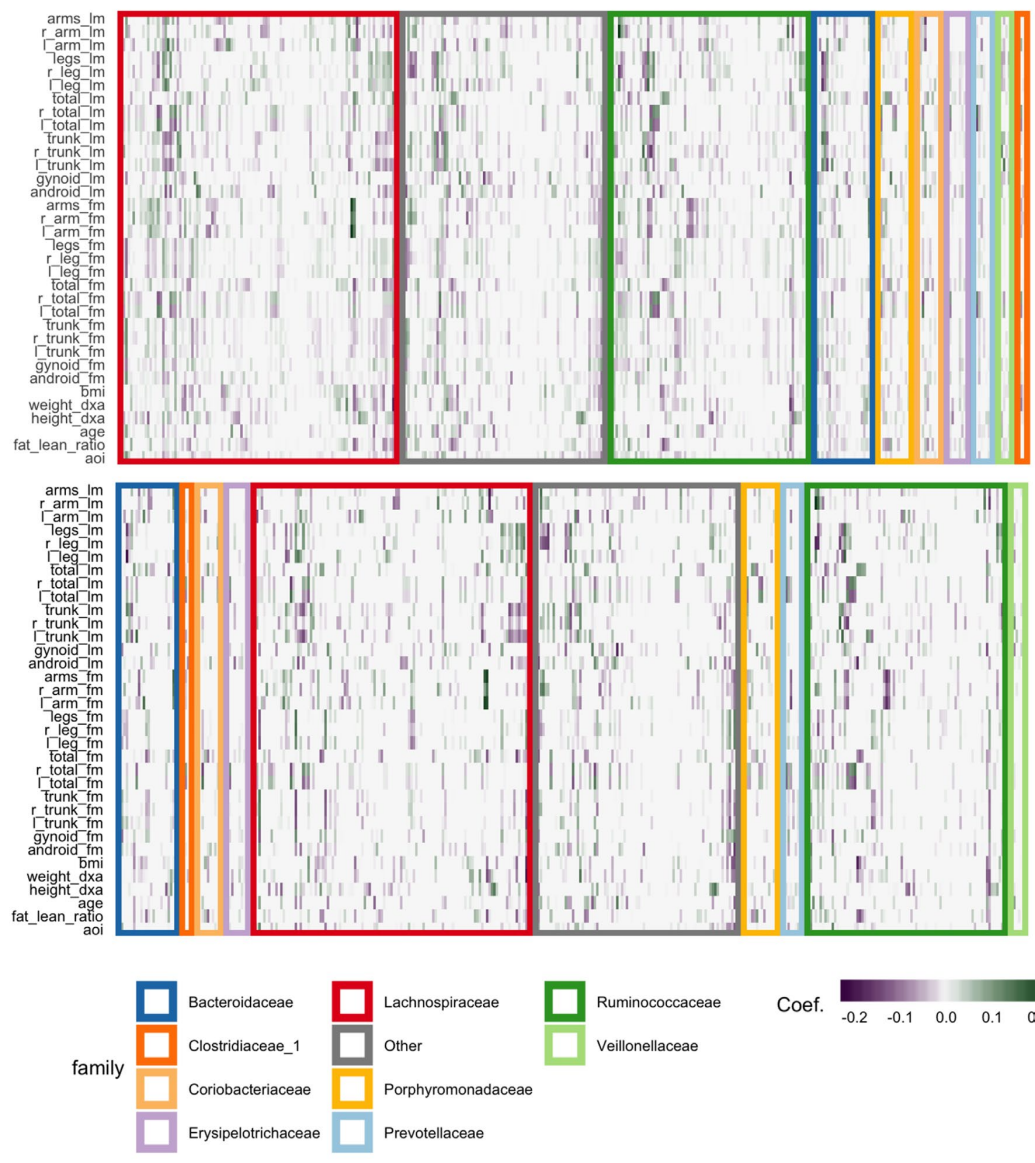
Coefficients for the graph-fused (top) and decoupled (bottom) lasso fits highlight groups of species with similar profiles across response variables. Colored rectangles demarcate taxonomic families. Individual cells give the coefficient for a particular species (column) for a given response variable (row). Purple and green denote negative and positive coefficients, respectively. Note that coefficient graph-fused panels have been smoothed according to correlation network between variables, as given in **Figure 11**. Species with similar coefficients are placed near one another. Note that even in the decoupled case, where there is no sharing across response problems, the coefficients nonetheless seem to be similar within lean and fat mass response groups, respectively. However, they are not as smooth as in the graph-fused lasso. As there is some consistency within these groups of variables, the form of structured regularization imposed by the graph seems appropriate.

The fitted coefficients from the graph-fused lasso are given in the top panel of **Figure 12**. The analogous display when the problem is decoupled into parallel lasso regressions is given in the bottom panel of the same figure.

Generally, both approaches highlight the same directions and size of association between individual species and the response variables, though those returned by the graph-fused lasso are smoother across responses. This smoothing may obscure true variation—for example, the stronger association between height_dxa and a few Ruminoccocus species—that appears in the parallel-lasso approach. On the other hand, regularization reduces the number of one-off nonzero coefficients, which are likely just noise.

There appear to be real associations between Lachnospiraceae and Ruminococcaceae and the body composition measurements. The strongest negative association between species abundance and fat mass occurs among a few species of Ruminococcaceae. Most species that have any association tend to have the same direction and magnitude of association across all body composition variables, not just those restricted to one mass type. This seems to be the case even in the parallel-lasso context, where such structure has not been directly imposed.

## Discussion

In this work, we have studied the problem of multitable data analysis, reviewing both the algorithmic foundations and practical applications of various methods. We have described approaches that are usually confined to particular literature areas and highlighted certain similarities in the process—for example, PCA-IV (section PCA-IV) and the graph-fused lasso (section Graph-Fused Lasso) were proposed in very different contexts, but have similar goals. By writing short, self-contained descriptions of various methods, we hope to contribute to an effort to distill ideas from the wide multitable data analysis literature to make them easily understandable to researchers interested in entering this field and useful for scientists hoping to apply these methods. A “cheat-sheet” summarizing some of the key properties of these methods is given in **Table 1**, and relevant packages can be found in **Table 2**.

**Table 1:** A high-level comparison of the multitable analysis methods discussed in this review. The purpose of this table is to give rules-of-thumb that can guide practical application, where choices invariably depend on the scale and structure of the data, the goals of the analysis, the expected number of future workflow applications, and availability of programming computation time.

Property	Algorithms	Consequence
Analytical solution	Concat. PCA, CCA, CoIA, MFA, PTA, Statico/Costatis	Methods with analytical solutions generally run much faster than those that require iterative updates, optimization, or Monte Carlo sampling. They tend to be restricted to more classical settings, however.
Require covariance estimate	Concat. PCA, CCA, CoIA, MFA, PTA, Statico/Costatis	Methods that require estimates of covariance matrices cannot be applied to data with more variables than samples, and become unstable in high-dimensional settings.
Sparsity	SPLS, Graph-Fused Lasso, Graph-Fused Lasso	Encouraging sparsity on scores or loadings can result in more interpretable, results for high-dimensional data sets. These methods provide automatic variable selection in the multitable analysis problem.
Tuning parameters	*Sparsity*: Graph-Fused Lasso, PMD, SPLS *Number of Factors*: PCA-IV, Red. Rank Regression, Mixed-Membership CCA Prior *Parameters*: Mixed- Membership CCA, Bayesian Multitask Regression	Methods with many tuning parameters are often more expressive than those without any, since it makes it possible to adapt to different degrees of model complexity. However, in the absence of automatic tuning strategies, these methods are typically more difficult to use effectively.
Probabilistic	Mixed-Membership CCA, Bayesian Multitask Regression	Probabilistic techniques provide estimates of uncertainty, along with representations of cross-table covariation. This comes at the cost of more involved computation and difficulty in assessing convergence.
Not Normal or Nonlinear	CCpNA, Mixed-Membership CCA, Bayesian Multitask Regression	When data are not normal (and are difficult to transform to normality) or there are sources of nonlinear covariation across tables, it can be beneficial to directly model this structure.
>2 Tables	Concat. PCA, CCA, MFA, PMD	Methods that allow more than two tables are applicable in a wider range of multitable problems. Note that these are a subset of the cross-table symmetric methods.
Cross-Table Symmetry	Concat. PCA, CCA, CoIA, Statico/Costatis, MFA, PMD	Cross-table symmetry refers to the idea that some methods don’t need a supervised or multitask setup, where one table contains response variable and the other requires predictors. The results of these methods do not change when the two tables are swapped in the method input.

**Table 2:** Pointers to R package that can be used to implement methods discussed in this survey. The vignettes in these packages go into more depth on the capabilities of these packages than do the short scripts used in our case study, available at https://github.com/krisrs1128/multitable_review.

Package	Methods	Documentation	Link
ade4	PCA, CCA, CoIA, Statico, Costatis, PCA-IV	Average	https://cran.r-project.org/web/packages/ade4/
FactoMineR	PCA, MFA	High	https://cran.r-project.org/web/packages/FactoMineR/
vegan	CCA, CCpnA	High	https://cran.r-project.org/web/packages/vegan/
spls	SPLS	High	https://cran.r-project.org/web/packages/spls/
PMA	PMD	High	https://cran.r-project.org/web/packages/PMA/
pls	PLS	High	https://cran.r-project.org/web/packages/pls/
Base R	PCA, CCA	High	https://cran.r-project.org/
GFLasso	Graph-Fused Lasso	Low	https://github.com/krisrs1128/gflasso
bayesMult	Bayesian Multitask Regression	Low	https://github.com/krisrs1128/bayesmult

In developing our WELL-China case study, we have both 1) described the types of interpretations facilitated by different approaches and 2) provided accessible implementations that can be incorporated into practical scientific workflows. Though our focus on a single application has allowed side-by-side comparisons of methods, we do not want to leave the reader with the impression that these methods are tied in any way to this particular biological analysis task. Indeed, the value of mathematical abstractions is that they can be applied to situations outside the imaginations of the original method designers. For example, consider these potential use cases:

- *Microbiome and metabolites:* If we replace the body composition table with the concentrations of different metabolites across samples, we can begin to make claims about covariation between microbiome community composition and host metabolic processes (Chong and Xia, 2017; Fukuyama et al., 2017).
- *Microbiome and metagenomics:* In addition to a species composition matrix, we might have data quantifying the presence of various genes. The methods in this review could be used to understand the relationship between community composition and functional capacity (Gill et al., 2006; Kurokawa et al., 2007).
- *Microbiome and perturbations:* If we had a matrix tracking the application of various perturbations to the host—the use of various medications, for example—we could use multitable methods to describe ways these (multidimensional) perturbations are related to microbiome community structure (Dethlefsen and Relman, 2011).

Our case study includes carefully thought-through visualizations of model results, a step that is crucial in scientific study but often overlooked in methodological research, where model results are reduced to tables of performance metrics. Recognizing that a good deal of effort in statistical work goes into data preparation and visualization of model results, we have ensured that codes for all steps are available, so that our work is fully reproducible.

We have found that multitable data analysis problems have motivated a wide range of analysis approaches. This is not surprising, considering the variety of contexts in which it arises, and it speaks to the richness of this methodological problem. As new data sources arise and as science evolves, we expect these ideas will inspire future generations of multitable research advances.

## Author Contributions

SH and KS conceived and designed the review, drafted the manuscript, and prepared all figures. KS implemented code for data analysis.

## Funding

KS was supported by a Stanford University Weiland fellowship and the National Institutes of Health T32 grant 5T32GM096982-04. SH is supported by the National Institutes of Health TR01 grant AI112401.

## Conflict of Interest Statement

The authors declare that the research was conducted in the absence of any commercial or financial relationships that could be construed as a potential conflict of interest.

## Footnotes

1 Though sampling is still ongoing.

2 Geometrically, the angle between original columns and the subspace, in the sense of **Figure 4**.

3 We are using 〈A, B〉 = tr(*A^T^B*).

4 Specifically, ${\left\|\left(\begin{array}{l} \beta_{\mathrm{android}}\\ \beta_{\mathrm{gynoid}} \end{array}\right)\right\|}_{2}>0.065.$

## Appendix

This appendix includes derivations and technical discussion of several methods surveyed in the main text: PCA-IV, PTA, and the C&W algorithm. While these methods can be understood and applied based on their computational description, these mathematical discussions provide motivation and context for their particular form.

### Derivation Details for PCA-IV

In this section, we provide the argument for why the generalized eigendecomposition ${\hat{\sum }}_{\mathrm{XY}}{\hat{\sum }}_{\mathrm{YX}}={\hat{\sum }}_{\mathrm{XX}}V\Lambda V^{T}$ provides the optimal V used in PCA-IV.

First consider *k* = 1. For any $\tilde{v}$, the objective in equation (5) has the form

(12) $$\begin{array}{c} \text{tr}\left({\hat{\Sigma }}_{\mathrm{YX}}\tilde{v}{\left(\tilde{v}{\hat{\Sigma }}_{\mathrm{XX}}\tilde{v}\right)}^{-1}{\left({\hat{\Sigma }}_{\mathrm{YX}}\tilde{v}\right)}^{T}\right)=\frac{{\tilde{v}}^{T}{\hat{\Sigma }}_{\mathrm{XY}}{\hat{\Sigma }}_{\mathrm{YX}}\tilde{v}}{{\tilde{v}}^{T}{\hat{\Sigma }}_{\mathrm{XX}}\tilde{v}}\\ =\frac{{\tilde{w}}^{T}\sum_{\mathrm{XX}}^{\frac{1}{2}}\sum_{\mathrm{XY}}^{-\frac{1}{2}}\sum_{\mathrm{YX}}\sum_{\mathrm{YX}}\sum_{\mathrm{XX}}^{-\frac{1}{2}}\tilde{w}}{\text{||}\tilde{w}|{|}_{2}^{2}} \end{array}$$

where we change variables $\tilde{w}=\sum_{\mathrm{XX}}^{\frac{1}{2}}\tilde{v}$. But to maximize equation (12), just choose $\tilde{w}$ to be the top eigenvector of $\sum_{\mathrm{XX}}^{-\frac{1}{2}}\sum_{\mathrm{XY}}\sum_{\mathrm{YX}}\sum_{\mathrm{XX}}^{-\frac{1}{2}}$, which implies that $\tilde{v}$ is the top generalized eigenvector of Σ*_XY_* Σ*_YX_* with respect to Σ*_XX_*. Indeed, in this case,

$$\begin{array}{c} \sum_{\mathrm{XY}}\sum_{\mathrm{YX}}\tilde{v}=\sum_{\mathrm{XY}}\sum_{\mathrm{YX}}\sum_{\mathrm{XX}}^{-\frac{1}{2}}\tilde{w}\\ =\sum_{\mathrm{XX}}^{\frac{1}{2}}\sum_{\mathrm{XX}}^{-\frac{1}{2}}\sum_{\mathrm{XY}}\sum_{\mathrm{YX}}\sum_{\mathrm{XX}}^{-\frac{1}{2}}\tilde{w}\\ =\sum_{\mathrm{XX}}^{\frac{1}{2}}\lambda_{1}\tilde{w}\\ =\lambda_{1}\sum_{\mathrm{XX}}\tilde{v}. \end{array}$$

Hence, in the case *K* = 1, the criterion is maximized by the top generalized eigenvector. For larger *K*, recall that the problem of maximizing $\frac{v^{T}\mathrm{Av}}{\text{||}v{\text{||}}^{\text{2}}}$ over *v* subject to being orthogonal to the first *K* − 1 eigenvectors of *A* is solved by the *K* ^th^ eigenvector of *A*, and applying this fact in step 12 of the argument above gives the result for general *K*.

### Derivation OF PTA *α*

The Lagrangian of the optimization defined by PTA is

$$ℒ(\alpha ,\lambda )=\sum_{l=1}^{L}\alpha_{l}\langle \bar{X},X.._{l}\rangle +\lambda (\text{||}\alpha {\text{||}}_{2}^{2}-1),$$

Which, when differentiated with respect to *α*, yields $\alpha_{l}=-\frac{1}{2\lambda }\langle \bar{X},X.._{l}\rangle$ for all *l*. The constraint that $||\alpha |{|}_{2}^{2}=1$ implies that $\frac{1}{4\lambda^{2}}{\sum_{l^{\prime }=1}^{L}\langle \bar{X},X.._{l^{\prime }}\rangle }^{2}=1$, which gives $\lambda =\frac{1}{2}\sqrt{\sum_{l^{\prime }=1}^{L}{\langle \bar{X},X.._{l^{\prime }}\rangle }^{2}}$, so $\alpha_{l}=\frac{\langle \bar{X},X.._{l}\rangle }{\sqrt{\sum_{l^{\prime }=1}^{L}{\langle \bar{X},X.._{l^{\prime }}\rangle }^{2}}}$.

### Derivation of Curds & Whey Shrinkage

Consider prediction across many related response variables. One way to pool information across responses is to define new fitted values from a linear combination of independent OLS fits. That is, to predict a response $y_{i}\in {\mathbb{R}}^{p_{1}}$, we set ${\hat{y}}_{i}^{\mathrm{cw}}=B{\hat{y}}_{i}^{\text{ols}}$ for some square matrix $B\in {\mathbb{R}}^{p_{1}\times p_{1}}$. But how to choose *B*?

One reasonable idea is to choose a *B* that has the best performance in a generalized cross-validation (GCV). The GCV approximation is that the *h_ii_* can be approximated by their average across all diagonal elements of *H*: $h_{\mathrm{ii}}\approx h:=\frac{1}{n}\text{tr (}H\text{)}$ for all *i*. In this spirit, define $g=\frac{1}{1-h}$ and approximate

$${\hat{y}}_{-1}\approx (1-g)y_{i}+g{\hat{y}}_{i}$$

Then, the leave-one-out CV error can be simplified to

$$\sum_{i=1}^{n}||y_{1}-B{\hat{y}}_{-i}{||}_{2}^{2}=\sum_{i=1}^{n}||y_{i}-B((1-g)y_{i}+g{\hat{y}}_{-i}{||}_{2}^{2},$$

and differentiating with respect to *B*, we find that the optimal ${\hat{B}}^{\text{cw}}$ in this GCV framework must satisfy

$$\sum_{i=1}^{n}(y_{i}-B((1-g)y_{i}+g{\hat{y}}_{-i})){\left((1-g)y_{i}+g{\hat{y}}_{-i}\right)}^{T},$$

or equivalently

$$\sum_{i=1}^{n}y_{i}{\left((1-g)y_{i}+g{\hat{y}}_{-i}\right)}^{T}=\sum_{i=1}^{n}B((1-g)y_{i}+g{\hat{y}}_{-1}){\left((1-g)y_{i}+g{\hat{y}}_{-1}\right)}^{T},$$

which in matrix form is

(13) $$(1-g)Y^{T}Y+g{\hat{Y}}^{T}Y=B{\left(\left(1-g)Y_{i}+g\hat{Y}\right)\right)}^{T}\left(\left(1-g)Y_{i}+g\hat{Y}\right)\right),$$

where $\hat{Y}\in {\mathbb{R}}^{n\times p_{1}}$ has *i* ^th^ row ${\hat{y}}_{-i}$.

Next, we can represent these cross-products in a way that is suggestive of CCA,

$$\begin{array}{c} Y^{T}Y=n{\hat{\sum }}_{\mathrm{YY}}\\ {\hat{Y}}^{T}Y=Y^{T}\mathrm{HY}=Y^{T}X{\left(X^{T}X\right)}^{-1}X^{T}Y=n{\hat{\sum }}_{\mathrm{YX}}{\hat{\sum }}_{\mathrm{XX}}^{-1}{\hat{\sum }}_{\mathrm{XY}}\\ {\hat{Y}}^{T}\hat{Y}=Y^{T}P_{X}^{2}Y=Y^{T}P_{X}Y=n{\hat{\sum }}_{\mathrm{YX}}{\hat{\sum }}_{\mathrm{XX}}^{-1}{\hat{\sum }}_{\mathrm{XY}}, \end{array}$$

Substituting this into equation (13) and ignoring the scaling *n* yields

$$\begin{array}{l} (1-g){\hat{\sum }}_{\mathrm{YY}}+g{\hat{\sum }}_{\mathrm{YX}}{\hat{\sum }}_{\mathrm{XX}}^{-1}{\hat{\sum }}_{\mathrm{XY}}=\\ B\left[(-g){\hat{\sum }}_{\mathrm{YY}}+(2g-g^{2}){\hat{\sum }}_{\mathrm{YX}}{\hat{\sum }}_{\mathrm{XX}}^{-1}{\hat{\sum }}_{\mathrm{XY}}\right]. \end{array}$$

Postmultiplying by ${\hat{\sum }}_{\mathrm{YY}}$ gives

(14) $$(1-g)I_{p1}+g{\hat{Q}}^{T}=B[(1-g)I_{p1}+(2g-g^{2}){\hat{Q}}^{T}],$$

where

$$\hat{Q}:={\hat{\sum }}_{\mathrm{YY}}^{-1}{\hat{\sum }}_{\mathrm{YX}}{\hat{\sum }}_{\mathrm{XX}}^{-1}{\hat{\sum }}_{\mathrm{XY}}\in {\mathbb{R}}^{P_{1}\times P_{1}}$$

Now, we claim that we can decompose $\hat{Q}=VD^{2}V^{-1}$, where $V\in {\mathbb{R}}^{p_{1}\times p_{1}}$ is the full matrix of CCA response directions and *D* is diagonal with the canonical correlations. Indeed, the usual CCA response directions *V* can be recovered by setting $V={\hat{\sum }}_{\mathrm{YY}}^{-\frac{1}{2}}\tilde{V}$, where $\tilde{V}$ comes from the SVD of $A:=\sum \frac{-\frac{1}{2}}{\mathrm{XX}}\sum_{\mathrm{XY}}\sum \frac{-\frac{1}{2}}{\mathrm{XX}}=\tilde{U}D{\tilde{V}}^{T}$. Hence

$$\begin{array}{c} Q=\sum_{\mathrm{YY}}^{{-}_{2}^{1}}A^{T}A\Sigma_{\mathrm{YY}}^{\frac{1}{2}}\\ =\sum_{\mathrm{YY}}^{{-}_{2}^{1}}{\tilde{V}}^{2}D^{2}{\tilde{V}}^{T}\Sigma_{\mathrm{YY}}^{\frac{1}{2}}\\ =VD^{2}V^{-1}, \end{array}$$

where we are able to write $V^{-1}={\tilde{V}}^{T}\sum_{\mathrm{YY}}^{-\frac{1}{2}}$ because $\tilde{V}$ is the full (untruncated) matrix of eigenvectors, so $\tilde{V}{\tilde{V}}^{T}=I$ in addition to the usual ${\tilde{V}}^{T}\tilde{V}=I$, which holds even for the truncated SVD.

Therefore, equation (14) can be expressed as

$$V^{-T}[(1-g)]I_{p_{1}}+gD^{2}]V^{T}=BV^{-T}[(1-g)]I_{p_{1}}+(2g-g^{2})D^{2}]V^{T}$$

and the *B* satisfying the normal equations has the form

$${\hat{B}}^{\text{cw}}=V^{-T}\Lambda \mathrm{VT},$$

where Λ is a diagonal matrix with entries

$$\lambda_{\mathrm{jj}}=\frac{1-g+d_{\mathrm{jjg}}^{2}}{1-g+(2g-g^{2})d_{\mathrm{jj}}^{2}}$$

Notice that when *n* is large, $\frac{1}{n}\text{tr }P_{X}$ will be small, leading to a smaller *g* ≈ 0 and less shrinkage. Recall that ${\hat{B}}^{\text{cw}}$ is used to pool across OLS fits, ${\hat{y}}_{i}^{\text{cw}}={\hat{B}}^{\text{cw}}{\hat{y}}_{i}^{\mathrm{ols}}$. That is,

$${\hat{Y}}^{\text{cw}}={\hat{Y}}^{\text{ols}}B^{T}={\hat{Y}}^{\text{ols}}V\Lambda V^{-1}$$

which we can also view as ${\hat{Y}}^{\text{cw}}V=\left({\hat{Y}}^{\text{ols}}V\right)\Lambda$. This means that the C&W coordinates along the canonical directions *V* are set as the OLS fits ${\hat{Y}}^{\text{ols}}$ along the canonical directions *V*, with weights defined by Λ. The actual ${\hat{Y}}^{\text{cw}}$ are recovered by transforming back to the original coordinate system. A similar way to view the C&W fits is to note ${\hat{Y}}^{\text{cw}}V=P_{X}(\mathrm{YV})\Lambda$, which is the original data *Y* according to the canonical directions, then projects the shrunk data onto the subspace defined by the columns of *X*. In any case, we see that C&W pools across regression problems through a soft shrinkage weighted along canonical response directions.
